# *Staphylococcus aureus* Interferes with Streptococci Spatial Distribution and with Protein Expression of Species within a Polymicrobial Oral Biofilm

**DOI:** 10.3390/antibiotics10020116

**Published:** 2021-01-26

**Authors:** Etyene Schnurr, Pune N. Paqué, Thomas Attin, Paolo Nanni, Jonas Grossmann, Silva Holtfreter, Barbara M. Bröker, Christian Kohler, Binh An Diep, Apoena de Aguiar Ribeiro, Thomas Thurnheer

**Affiliations:** 1 Instituto de Saúde de Nova Friburgo, Federal Fluminense University, 28625-650 Nova Friburgo, Brazil; 2 Clinic of Conservative and Preventive Dentistry, Center of Dental Medicine, University of Zurich, 8032 Zurich, Switzerland; punenina.paque@zzm.uzh.ch (P.N.P.); thomas.attin@zzm.uzh.ch (T.A.); thomas.thurnheer@zzm.uzh.ch (T.T.); 3 Functional Genomics Center, ETH Zürich and University of Zurich, 8057 Zurich, Switzerland; paolo.nanni@fgcz.uzh.ch (P.N.); jonas.grossmann@fgcz.uzh.ch (J.G.); 4 SIB Swiss Institute of Bioinformatics, 1015 Lausanne, Switzerland; 5 Department of Immunology, University Medicine Greifswald, 17475 Greifswald, Germany; silva.holtfreter@med.uni-greifswald.de (S.H.); broeker@uni-greifswald.de (B.M.B.); 6 Friedrich-Loeffler Institute for Medical Microbiology, University Medicine Greifswald, 17475 Greifswald, Germany; kohlerc@uni-greifswald.de; 7 Division of HIV, Infectious Diseases, and Global Medicine, Department of Medicine, University of California San Francisco, San Francisco, CA 94143, USA; binh.diep@ucsf.edu; 8 Division of Diagnostic Sciences, University of North Carolina, Chapel Hill, NC 27599, USA; apoena@email.unc.edu

**Keywords:** multi-species biofilm, *Streptococcus oralis*, *Streptococcus mutans*, proteomic analysis, MSCRAMM, *Staphylococcus aureus*

## Abstract

We asked whether transient *Staphylococcus aureus* in the oral environment synergistically interacts with orally associated bacterial species such as *Actinomyces oris*, *Candida albicans*, *Fusobacterium nucleatum*, *Streptococcus oralis*, *Streptococcus mutans*, and *Veillonella dispar* (six-species control biofilm 6S). For this purpose, four modified biofilms with seven species that contain either the wild type strain of the *S. aureus* genotype (USA300-MRSA WT), its isogenic mutant with MSCRAMM deficiency (USA300-MRSA ΔMSCRAMM), a methicillin-sensitive *S. aureus* (ST72-MSSA-) or a methicillin-resistant *S. aureus* (USA800-MRSA) grown on hydroxyapatite disks were examined. Culture analyses, confocal-laser-scanning microscopy and proteome analyses were performed. *S. aureus* strains affected the amount of supragingival biofilm-associated species differently. The deletion of MSCRAMM genes disrupted the growth of *S. aureus* and the distribution of *S. mutans* and *S. oralis* within the biofilms. In addition, *S. aureus* caused shifts in the number of detectable proteins of other species in the 6S biofilm. *S. aureus* (USA300-MRSA WT), aggregated together with early colonizers such as *Actinomyces* and streptococci, influenced the number of secondary colonizers such as *Fusobacterium nucleatum* and was involved in structuring the biofilm architecture that triggered the change from a homeostatic biofilm to a dysbiotic biofilm to the development of oral diseases.

## 1. Introduction

*Staphylococcus aureus* (*S. aureus*) colonizes the skin or mucous membranes of a broad range of hosts, including humans, and is also responsible for an enormous burden on the health care system [1]. As an opportunistic pathogen, *S. aureus* mainly infects individuals who have medical device implants, suffer from barrier dysfunctions or are immunocompromised. These versatile bacteria can cause a large spectrum of diseases, including osteomyelitis, endocarditis, wound infections, pneumonia, and sepsis [1,2]. One of the reasons that staphylococci can successfully infect human tissues is their ability to attach to surfaces and develop into recalcitrant community structures known as “biofilms”. The development of biofilms has been considered important in many types of infections and they represent a clinical challenge as they are highly resistant to antimicrobial therapies and often occur in areas of the body that are not easily accessible for treatment [3].

Generally, oral biofilms are present on all intra-oral surfaces. They are composed of multi-species microorganisms that interact with and maintain a mutually beneficial relationship with each other and with the host. Physical proximity plays a role in these interactions, which can be synergistic or antagonistic [4]. Although oral microorganisms exist in symbiosis, some can turn into pathogens that cross the barrier of commensalism, leading to the interruption of homeostasis, or “dysbiosis” [5]. The functional aspects that shift a biofilm to dysbiosis remain largely unknown [6]. What is clear, however, is that oral diseases arise as a result of a change in the proportion of certain species, with greater pathogenic potential in the indigenous flora, accompanied by the host’s immune reaction and an inflammatory response. Therefore, it is the prevalence of a certain combination of microbial species associated with the host’s inability to contain its proliferation that is more indicative of a risk for disease development.

The in vitro polymicrobial biofilm model is a tool used to study cell–cell communication and species behaviour. For example, *S. aureus* [7] and *Candida albicans* (*C. albicans*) [6,8,9] are well integrated in the oral biofilm model, being associated with the growth of organisms, such as *Fusobacterium nucleatum* (*F. nucleatum*) and *Pseudomonas aeruginosa*, respectively. In order to generate a three-dimensional, non-destructive visualization of both Gram-positive and Gram-negative bacteria, these in vitro biofilms can be combined with the fluorescent in situ hybridization (FISH) technique using probes to target specific 16S rRNA sequences and confocal-laser-scanning microscopy (CLSM) [10].

The oral biofilm pathogenesis can also be influenced by the structural and biochemical components of the extracellular matrix. Both the extracellular polysaccharide intercellular adhesion (PIA) [11] and the proteins anchored in the cell wall, which are linked by a covalent bond (cell-wall-anchored (CWA) proteins) [12], play a role in the formation of biofilms [13]. *S. aureus* CWA is classified into four groups based on their structural motifs: the MSCRAMM (component of the microbial surface that recognizes adhesive matrix molecules), the NEAT motif family, the helical triple-beam family, and the G5-E repeat family. Here, we focus on MSCRAMM because they not only promote the interaction between pathogens and tissues, but also provide ingenious strategies for bacterial evasion from the host’s immune response [12,14]. In the case of *S. aureus*, MSCRAMM (fibronectin-binding proteins, clumping factors, serine aspartate repeats proteins, and collagen-binding proteins) are important surface proteins expressed in vivo during infection and represent new immune-therapeutic targets [15]. The clumping factor (clfA) has been shown to inhibit fibrinogen binding to platelets and fibrinogen-dependent platelet aggregation, indicating that its binding site occludes the platelet binding site [16,17]. In murine *S. aureus* septicemia [18] and in the abscess model [19], mice without the clfA-binding fibrinogen motif showed better survival rates, suggesting that the clfA–fibrinogen interactions in blood contribute to virulence. In addition, a clfA mutant in *S. aureus* had a reduced ability to cause vegetation in a model of endocarditis in rats [20].

Global changes in gene regulation occur throughout the life cycle of staphylococcal biofilms and are strain-specific. The biofilm life cycle comprises three stages: adhesion (adhesion, fixation), aggregation (maturation, accumulation), and detachment (dispersion). Moreover, biofilms seem to be produced by distinct mechanisms in methicillin-resistant *S. aureus* (MRSA) and methicillin-sensitive *S. aureus* (MSSA) [21]. MSSA strains commonly produce an *icaADBC* operon-encoded polysaccharide intercellular adhesin (PIA)-dependent biofilm. In contrast, the release of extracellular DNA (eDNA) and cell-surface expression of a number of sortase-anchored proteins, and the major autolysin have been implicated in the biofilm phenotype of MRSA isolates [21].

Previously determined data indicate that *S. aureus* proteins and virulence factors are important in biofilm formation, but a clear picture of their influence in a polymicrobial environment that mimics the oral conditions has yet to emerge. Therefore, the aim of this study was to provide a comprehensive analysis of the formation of staphylococcal biofilms interacting with a variety of species found in the oral cavity (*Actinomyces oris*, *C. albicans*, *F. nucleatum*, *S. oralis*, *S. mutans*, and *Veillonella dispar*). Emphasis is placed on the expression and regulation of adhesins and on the proteomic dynamics of staphylococcal communities within oral multispecies biofilms. In addition, the spatial skeletal structure of the oral biofilm in the presence of staphylococci will be analyzed to deepen the understanding of the benign and pathogenic characteristics of *S. aureus*.

## 2. Experimental Section

### 2.1. Bacterial Strains and Growth Conditions

*S. aureus* strains (Table 1) were selected based on clinical relevance and genotype, including multilocus sequence type (MLST) [22], spa type [23], agr type, and staphylococcal cassette chromosome (SCC) mec type, and the presence or absence of the Panton–Valentine leukocidin (PVL)-encoding genes (lukF-PV and lukS-PV). Virulence and resistance genes were detected using the StaphID DNA microarray (Alere Technologies GmbH, Jena, Germany) as previously described [24]. MLST and spa typing was performed according to published standard protocols [25,26]. Antibiotic resistance was determined using the Vitek2 system with AST-P632 cards (bioMérieux, Nürtingen, Germany), as previously reported [27]. Detailed information of the genotype, virulence gene profile, and antibiogram of PN35 and HU13N clinical isolates are described in Supplemental Table S1. The SF8300, a prototypical USA300-0114 genotype wild-type strain [28], was used as a background for the sequential mutant derivative generated in the present work, i.e., (USA300- MRSA ΔMSCRAMM), with allelic replacement of seven genes encoding the microbial surface components recognizing the adhesive matrix molecules (clfA, clfB, sdrC, sdrD, sdrE, fnBPA and fnBPB) (see Section 2.2.). *S. aureus* strains were routinely cultivated in air supplemented with 5% CO_2_ at 37 °C for 3 days unless otherwise stated. For this, Columbia blood agar (CBA) plates (containing 5% defibrillated human blood, 5 mg hemin/l, 10 mg Vitamin K/l, Columbia agar base), or tryptic soy agar plates or tryptic soy broth (TSB), supplemented with 0.6% yeast extract, and 0.8% glucose (Difco) were used.

### 2.2. Construction of the Staphylococcus Aureus Mutant Strain

In-frame deletion in SF8300 was performed using the pKOR1 allelic replacement mutagenesis system, as described previously [31,32]. The ΔMSCRAMM strain (USA300-MRSA ΔMSCRAMM) was constructed by sequential, in-frame deletions of *clfA*, *clfB*, *sdrC*, *sdrD*, *sdrE*, *fnbA* and *fnbB*. The primers used for the construction of in-frame deletions of *clfA* and *clfB*, for the construction of the triple deletion of *sdrC-sdrD-sdrE* genes, and for double deletions of *fnbA* and *fnbB* genes are described in Supplemental Table S2. In brief, sequences flanking each of the MSCRAMM loci intended for deletion were PCR upstream (1000 bp) and downstream (1000 bp) amplified from SF8300 genomic DNA. PCR products were used for recombination with pKOR1, and the resulting plasmid, pKOR1-ΔMSCRAMM, was transferred by electroporation to *S. aureus* RN4220, and subsequently to SF8300. The deletion of each MSCRAMM was further confirmed by PCR and by DNA sequencing.

### 2.3. Multispecies Biofilm Formation and Harvesting

In addition to the *S. aureus* strains, the following six strains were used in this study: *Actinomyces oris* (OMZ 745), *Candida albicans* (OMZ 110), *Fusobacterium nucleatum subsp. nucleatum* (OMZ 598), *Streptococcus oralis* (OMZ 607), *Streptococcus mutans* (OMZ 918), and *Veillonella dispar* (OMZ 493). A six-species biofilm was cultivated as previously reported [33], and in the present work is referred to as the 6S control biofilm. Four modified seven-species biofilms were also developed in parallel, referred to as the 6S + USA300-MRSA WT (six-species biofilm + SF8300 (USA300) biofilm), 6S + USA300-MRSA ΔMSCRAMM (six-species biofilm + MSCRAMM-deficient *S. aureus*), 6S + ST2-MSSA (six-species biofilm + PN35), and 6S + USA800-MRSA (six-species biofilm + HU10L). Briefly, all strains were maintained on CBA plates. Prior to biofilm formation, precultures were produced by inoculating each strain in modified fluid universal medium (mFUM) for 16 h anaerobically. Then, 0.8–1.5 mL of the precultures were transferred to fresh medium and incubated for 8 h. The final precultures were mixed in equal volumes and densities (OD_550_ = 1.0 ± 0.05) to generate a mixed microbial suspension. Hydroxyapatite disks (HA; Ø 9 mm, Clarkson Chromatography Products, Inc., South Williams-port, PA, USA) were covered with pasteurized whole saliva from different individuals and incubated for 4 h at room temperature to form a pellicle [34]. To induce biofilm formation, 200 μL of the microbial suspension and growth medium 1.6 mL (1120 µL saliva + 480 µL mFUM + 0.3% glucose supplemented with Sørensen’s buffer at a final of pH 7.2) were loaded on the pellicle-coated disks and anaerobically incubated for 64 h. During the incubation, the cultivated medium was replenished at 16 and 40 h. The carbohydrate concentration of the growth medium was changed after 16 h, replacing the 0.3% glucose with 0.15% glucose and 0.15% sucrose. The biofilm disks were dip-washed in 0.9% *w*/*v* NaCl at 16, 20, 40, 44, 48, and 64 h. After 64 h, biofilms were harvested and either suspended in 0.9% *w*/*v* NaCl for culture analysis and proteomic analysis or fixed in 4% paraformaldehyde for image analysis.

### 2.4. Image Analysis with Confocal Laser Scanning Microscopy (CLSM)

The paraformaldehyde-fixed biofilms were stained by fluorescence in situ hybridization and subjected to CLSM for imagine analysis. In short, 64 h biofilms were fixed immediately with 4% (*w*/*v*) paraformaldehyde for 2 h at 4 °C, and permeabilized for 1 h at 37 °C by exposure to lysozyme solution (10 mg/mL (46,200 U/mL) in 98 mM Tris/HCl pH 7.5 and 5 mM EDTA) and 15 min to proteinase K (2 µg/mL in Tris/HCl pH 7.5 and EDTA). An extra 100 U/mL of mutanolysin was added for the permeabilization of streptococci. Biofilms were prehybridized in hybridization buffer (0.9 M NaCl, 20 mM Tris/HCl, 30% formamide, 0.01% SDS) at 46 °C for 15 min, followed by hybridization for 3 h using the same buffer containing fluorescently labelled oligonucleotide probes. Details of the probes used in this study are listed in Table 2. In short, *S. aureus* was marked with Saur229-Cy3, *S. mutans* with MUT590-Cy5, and *S. oralis* with MIT446-FAM. After the hybridization process, labelled biofilms were washed for 45 min at 48 °C to remove not specifically bound staining material and then counterstained using 400 µL of 4′,6-diamidino-2-phenylindole (DAPI 0.5 ng/µL in Nanopure H_2_O) for 20 min at room temperature in the dark. All samples were then embedded upside-down on chamber slides in Mowiol overnight at room temperature [33]. Confocal images were captured with a 100× objective (oil, NA 1.5, Leica Microsystems) on a Leica sp5 confocal microscope (Leica Microsystems, Wetzlar, Germany) [35]. The image acquisition was performed with a line average of 5 acceleration 1 and z sections of 1.02 µm. A UV laser (excitation 405 nm), an Argon laser (488 nm), a DPSS diode laser (561 nm), and a Helium-Neon laser (633 nm excitation) were applied and PTM detectors were used with a bandwidth of 413–474 nm to detect DAPI, to 501–555 nm for FAM, to 570–620 nm for Cy3, and to 660–710 nm for Cy5. The captured images were processed using Imaris software (version 7.4.0, Bitplane, Zurich, Switzerland) to reconstruct the biofilm.

### 2.5. Culture Analyses

The CFU counts were performed to quantify the numbers of individual species in different biofilm models. The respective biofilms disks were vortexed for 1 min in 1 mL of 0.9% NaCl and at 30 W for 5 s (Sonifier B-12, Branson Ultrasonic, Urdorf, Switzerland) to enable bacterial dispersal. A dilution of the biofilm suspension was performed to gain readable CFU counts of 20–200 per plate. Of each dilution series, 50 μL were plated on the agar using an EDDY Jet Auto Spiral Diluter (IUL Instruments, Barcelona, Spain)). For *S. oralis* and *S. mutans*, Difco™ mitis salivarius agar plates (Becton, Dickinson and Company, East Rutherford, NJ, USA) were used, after supplementation with 0.001% *w*/*v* sodium tellurite (BDH Chemicals Ltd, Poole, UK.). For the species-specific selection of *F. nucleatum,* fastidious anaerobe agar plates (Neogen) with 1 mg/L erythromycin (Sigma-Aldrich, St. Louis, MO, USA), 4 mg/L vancomycin (Sigma-Aldrich, St. Louis, MO, USA), and 1 mg/L norfloxacin were applied. *C. albicans* was detected on Biggy agar plates (Difco). The unspecific total microbial count was determined with Columbia blood agar plates (Oxoid), which were supplemented with 5% whole human blood. Each group was tested with nine biological replicates.

### 2.6. Bacterial and Biofilm Protein Extraction

Protein analysis was performed on the biofilm bacterial cell lysates from three different conditions (6 S control biofilm, 6S + USA300-MRSA WT strain, and 6S + USA300-MRSA ΔMSCRAMM strain using three biological replicates. Bacterial lysates for LC-MS/MS were compared as follows. Multispecies biofilm pellets for control, SF8300 biofilm, and ΔMSCRAMM biofilm were collected as previously described [37]. Samples were prepared for proteomics by using a commercial iST Kit (PreOmics, Martinsried, Germany) with an updated version of the protocol. Briefly, cell pellets were solubilized in ‘Lyse’ buffer, boiled at 95 °C for 10 min and processed with High Intensity Focused Ultrasound (HIFU) for 30 s setting the ultrasonic amplitude to 85%. The Qubit^®^ Protein Assay Kit (Life Technologies, Zurich, Switzerland) was then used to estimate the total protein concentration, of which 50 μg of proteins were mixed with the ‘Digest’ solution for 60 min at 37 °C. The digestion was stopped by adding the ‘Stop’ solution (100 μL) to the cartridge. A final centrifugation step (3800× *g*) was performed to remove the solutions and separate the peptides using the iST-filter. The LC-MS analysis was performed after further washing, eluting, drying, and resolubilizing steps in 15 µL of loading buffer (3% acetonitrile, 0.1% formic acid).

### 2.7. Liquid Chromatography-Mass Spectrometry Analysis

Mass spectrometry analysis was performed on a nanoAcquity UPLC (Waters Inc., Milford, MA, USA) connected to a Q Exactive mass spectrometer (Thermo Scientific, Waltham, MA, USA) equipped with a Digital PicoView source (New Objective). For each sample, 2 μL were injected. Peptides were trapped on a MZ Symmetry C18 Trap Column (100 Å, 5 µm, 180 × 20 mm, Waters Inc., Milford, MA, USA) and separated on a nanoEase MZ C18 HSS T3 Column (100 Å, 1.8 µm, 75 × 150 mm, Waters, Milford, MA, USA) at a flow rate of 300 nL/min using a gradient from 8% solvent B (0.1% formic acid in acetonitrile) to 22% solvent B within 79 min, 32% B in 11 min and 95% B in 10 min. The samples were acquired in a randomized order. The mass spectrometer settings were set for data-dependent analysis (DDA), as follow: full-scan MS in spectra in scan range 300–1700 *m*/*z*, resolution 70,000, after accumulation to a target value (AGC) of 3,000,000; fragmentation MS/MS spectra acquired on the twelve most intense signals per cycle, using higher-energy collision dissociation (HCD) fragmentation at a normalized collision energy of 25, resolution 35,000, maximum injection time of 120 ms and AGC value of 50,000. Singly and unassigned charge states were rejected. Only precursors with intensity above 2% under-fill ratio were selected for MS/MS. An exclusion list of 30 s was configured to exclude for MS/MS measurement previously selected precursor masses (10 ppm window). Internal lock mass calibration was performed using *m*/*z* 371,1010 and 445, 1200. The local laboratory information management system (LIMS) was used to handle the mass spectrometry proteomics data [38].

### 2.8. Protein Identification and Label-Free Quantification

The acquired raw MS data were processed by MaxQuant (version 1.4.1.2), followed by protein identification using the integrated Andromeda search engine [39]. Spectra were searched against a FASTA protein database containing Swissprot human sequences (UP000005640, v2018-02-23), common protein contaminants and Uniprot sequences from *Staphylococcus aureus USA300* (UP000001939, v2018-02-23); *Streptococcus mutans UA159/ATCC700610* (UP000002512, v2018-02-23); *Fusobacterium nucleatum subsp. Nucleatum* (UP000005392, v2018-02-23); *Fusobacterium nucleatum subsp. vicentini KP-F2* (UP000006454, v2018-02-23); *Veillonella dispar ATCC17748* (UP000003529, v2018-02-23); *Candida albicans* (UP000000559, v2018-02-23); *A. naeslundii str. Howell 279* (UP000007814, v2018-02-28); *Actinomyces sp. oral taxon 178 str. F0338* (UP000003389, v2018-02-28); *Actinomyces sp. oral taxon 448 str. F0400* (UP000005351, v2018-02-28); *Actinomyces sp_Oral_Taxon_175, str. F0384* (UP000004281 v2018-02-28), and decoy (‘revert’) sequences. Carbamidomethylation of cysteine was set as fixed modification, while methionine oxidation and N-terminal protein acetylation were set as variables. Enzyme specificity was set to trypsin/P, allowing a maximum of two missed cleavages and minimal peptide length of seven amino acids. The default MaxQuant Orbitrap search settings were used, with a peptide and protein maximum false discovery rates (FDR) of 0.01 and 0.05, respectively. The options for label-free quantification and the match between runs were enabled, allowing for a 2-min window. When setting up the MaxQuant experimental design template, the files are kept separate in order to obtain individual quantitative values. The Intensity values, reported in the proteinGroups.txt file, were used to compute the protein fold changes, and the R package SRMService [40] was used for further processing (filter for proteins with at least two peptides, a maximum of four missing values, data normalization using a modified robust z-score transformation, computation of p-values using the *t*-test with pooled variance). In the case of proteins missing measurements in one of the conditions, a pseudo fold change was calculated replacing the missing group average by the mean of 10% smallest protein intensities in that condition. The comparison was made between the different types of multispecies biofilm lysates (i.e., 1. 6S + USA300-MRSA WT versus 6S + USA300-MRSA ΔMSCRAMM; 2. 6S versus 6S + USA300-MRSA WT and 3. 6S versus 6S + USA300-MRSA ΔMSCRAMM). Quantified proteins a with significant raw p value (*p* < 0.05), at least two unique peptides, and an absolute value of log2 fold change ≥ 2 were considered as true regulated proteins. Proteomic data (Supplemental Data upon request) contain a heatmap for quantifiable protein sorted by missing and intensity (log2) and quality control with distribution of intensities and normalization.

### 2.9. Functional and Ontology Analysis

The role the 6S + USA300-MRSA WT biofilm and the 6S + USA300-MRSA ΔMSCRAMM biofilm were investigated using Gene ontology (GO) terms from regulated proteins. The lists of the GO were determined with Uniprot (released on May 2017), applying the “Retrieve/ID Mapping” function. Redundant terms were thereby removed, based on the REVIGO analyses (released on June 2018), applying “small (0.5)” similarities. A defined classification off gene nomenclatures (molecular functional, biology process, and cellular component) was applied to generate a GO list summary. GO terms were classified as “other”, if < 2% of the whole GO was detected in each domain.

### 2.10. Statistical Analysis

The microbial data from biofilm growth and CFU counting from the culture analyzes were analyzed using an unpaired *t*-test or a one-way ANOVA. A Bonferroni post hoc test was used for the latter, to compare between the individual groups (Prism v.6 GraphPad software). The level of significance was set at *p* < 0.05. The results are shown as the mean ± standard deviation (SD) or as box plots with median and interquartile ranges (IQR).

## 3. Results

### 3.1. S. Aureus Strains Differently Affect the Number of Supragingival-Associated Species Grown on Hydroxyapatite Disks

**Figure 1:**
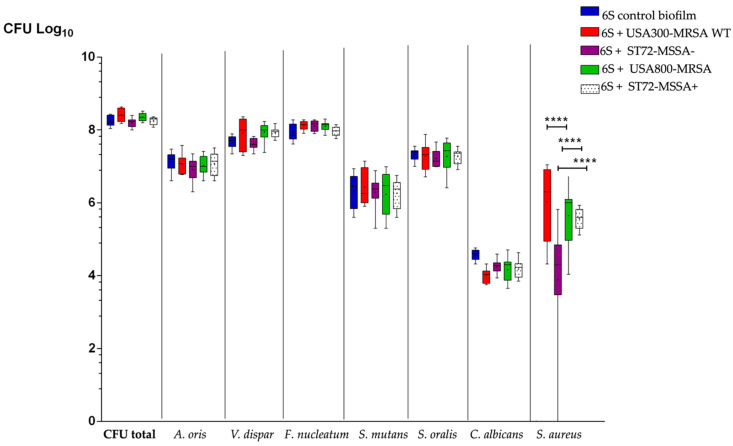
Boxplots with median and interquartile ranges present the impact of various *S. aureus* strains on the growth of supragingival-associated oral species. Quantification was performed using species-specific culture analysis. The 6S control biofilms are presented in blue, the 6S + USA300-MRSA WT (wild type *S. aureus*) strain in red, the 6S + ST72-MSSA (methicillin-sensitive *S. aureus* strain PN35, PVL negative) in purple, the 6S + USA800-MRSA (methillicin-resistant *S. aureus* HU13N, PVL negative) in green, and the MSSA-OMZ1122, PVL positive is presented in white/dotted. The data are expressed as the bacterial mean counts ± SD from nine biological replicates, presented on a log_10_ scale per milliliter. Highly significant differences were detected between USA300-MRSA WT and ST72-MSSA-, as well as between ST72-MSSA- and USA800-MRSA, and ST72-MSSA- and ST72-MSSA+ (ANOVA test: **** *p* < 0.0001). No significant differences were found between USA300-MRSA WT and USA800-MRSA, USA300-MRSA WT and ST72-MSSA+, and USA800-MRSA and ST72-MSSA+.

To test whether *S. aureus* strains differ in their ability to grow in a polymicrobial oral biofilm, we co-incubated six different oral bacterial species (6S) with different *S. aureus* isolates (*n* = 4), which varied in their resistance and virulence gene profile, on hydroxyapatite disks (Table 1). After anaerobic cultivation for 64 h at 37 °C, the numbers of the individual oral microbial species within the biofilms were estimated by culture analyses on selective agar plates (Figure 1).

All *S. aureus* strains were able to integrate well into the 6S biofilm model that contained *A. oris*, *V. dispar*, *F. nucleatum*, *S. mutans*, *S. oralis*, and *C. albicans* grown on hydroxyapatite disks. The presence of MSSA strains to the 6S biofilm showed only a slight impact on the CFU quantities of the oral biofilm species. *V. dispar* and *F. nucleatum* moderately increased in numbers when adding the WT or MRSA strain to the other microorganisms. Both strains (WT and MRSA) developed higher CFUs in the biofilm than the MSSA strains ST72-MSSA-, PN35 (PVL-negative) and ST72-MSSA+, OMZ1122, PVL-positive (*p* < 0.0001).

No difference was detected between the growth of the *pvl*-positive WT and the *pvl*-negative MRSA strain. However, higher counts were detected for the *pvl*-positive ST72-MSSA+, OMZ1122 strain when compared to the *pvl*-negative ST72-MSSA-, PN35 strain (Figure 1).

### 3.2. MSCRAMM Influence the Growth of S. aureus in Multispecies Oral Biofilms

**Figure 2:**
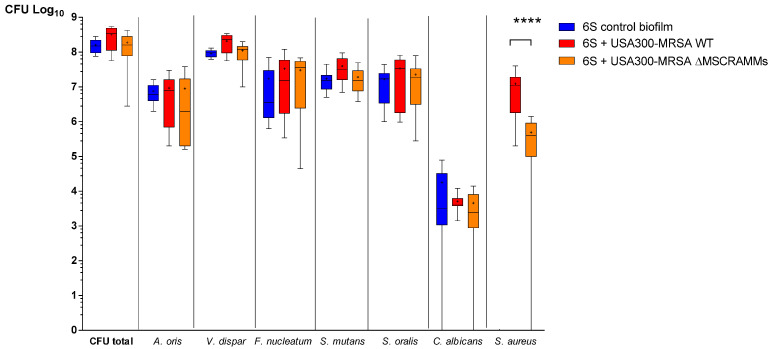
Boxplots with median and interquartile ranges showing the influence of *S. aureus* MSCRAMM on the growth of *S. aureus* in multispecies oral biofilms. Quantification was performed using species-specific culture analyses. The 6S control biofilms are presented in blue, the 6S + WT (implemented the wild type *S. aureus* strain) is presented in red, and the 6S+ ΔMSCRAMM (implemented the MSCRAMM-deficient *S. aureus* strain) in orange. The data are expressed as the mean ± SD of bacterial counts from 9 biological replicates, presented on a log_10_ scale per milliliter. ANOVA test: **** *p* < 0.0001.

MSCRAMM play a decisive role in the early phase of *S. aureus* biofilm formation. Therefore, we investigated the influence of MSCRAMM on the growth of *S. aureus* in the 6S biofilm setting. Adding the WT strain (SF8300 wild type) to the 6S increased the total number in CFU in the supragingival biofilm by a factor of 2 (Figure 2 **** *p* < 0.0001). However, when the USA300-MRSA ΔMSCRAMM strain was inoculated together with the 6S biofilm, this increase was no longer observed. Moreover, on a species-specific level, higher numbers were observed for *V. dispar* and streptococci microorganisms after co-incubation with the USA300-MRSA WT strain, but not with the USA300-MRSA ΔMSCRAMM mutant (Figure 2).

### 3.3. The Distribution of S. mutans and S. oralis is Modified When Staphylococcus Aureus MSCRAMM Genes are Deleted

**Figure 3:**
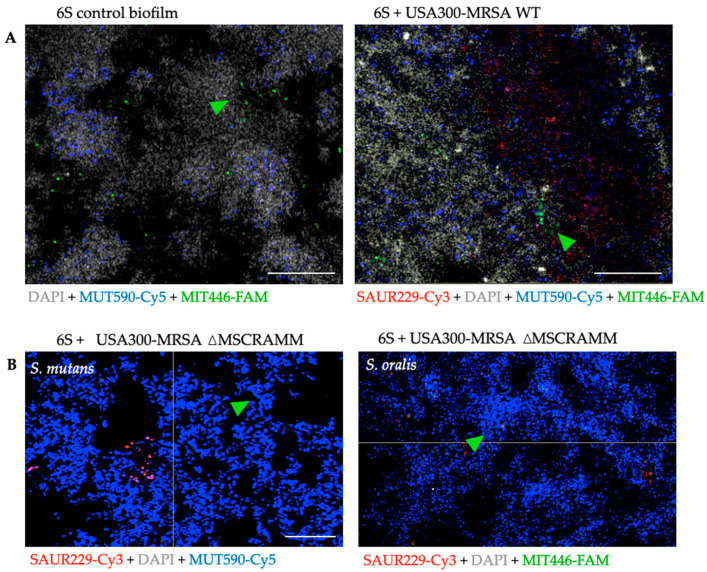
Localization of *S. mutans* and *S. oralis* within the biofilms. In (**A**), confocal-laser-scanning microscopy images of fluorescence in situ hybridization (FISH): biofilm cells shown in gray stained with DAPI, and *S. aureus* (USA300-MRSA WT and ΔMSCRAMM) stained with Saur229-Cy3 appear red. Due to MUT590-Cy5 staining, *S. mutans* appears blue and due to MIT446-FAM staining, *S. oralis* appears green. The thickness of the biofilm was 32.6 and 45.4 µm, respectively. In (**B**), both images show the 6S biofilm with the implemented USA300-MRSA ΔMSCRAMM strain, presenting biofilm cells in blue. Arrows detail the green staining for *S. mutans* (MUT590-FAM) and *S. oralis* (MIT446-FAM). The thickness of the biofilm was 17.1 and 16.1 µm. Scale = 20 µm.

Next, we investigated the spatial distribution of *S. aureus* along with the 6S biofilm by means of FISH staining and visualization with CLSM. As expected, USA300-MRSA WT cells appeared to be more abundant in the biofilms as compared to cells of USA300-MRSA ΔMSCRAMM. In addition, CLSM revealed apparent alterations regarding the general structural conformation of the whole biofilms with USA300-MRSA WT (Figure 3A). In this scenario, the localization of *S. mutans* and *S. oralis* within the biofilm structure was mostly clustered in the form of aggregates near *S. aureus* strains and mainly distributed on the “inner” biofilm side closer to the hydroxyapatite disk surface (Figure 3A). To determine if the preferred location of the streptococci was related to the quantity of *S. aureus* within the 6S biofilm given that the USA300-MRSA ΔMSCRAMM had a reduced growth number, we analyzed the distribution of *S. mutans* and *S. oralis* in the USA300-MRSA ΔMSCRAMM biofilm. Under this condition, the distribution of *S. mutans* and *S. oralis* with *S. aureus* aggregates was no longer observed (Figure 3B).

### 3.4. Staphylococcus Aureus Causes Shifts in the Numbers of Other Species’ Detectable Proteins in the Six-Species Biofilm

Next, we performed comparative proteomics on the total biofilm protein extracts obtained from the 6S control biofilm, the 6S + USA300-MRSA WT and the 6S + USA300-MRSA ΔMSCRAMM biofilms. The total numbers of proteins quantified were of 1579 in the 6S +WT versus the 6S + USA300-MRSA ΔMSCRAMM biofilm, 1570 in the 6S + USA300-MRSA WT versus 6S control biofilm, and 1555 in the in the 6S + USA300-MRSA ΔMSCRAMM versus the 6S control biofilm. For each of these comparisons, the proteins quantified in both type of samples were 99.68% (*n* = 1574), 96.49% (*n* = 1515), and 97.49% (*n* = 1516), respectively. Among these subsets, the data were screened for differently regulated proteins showing a log2 fold change of |1| in one sample type (*p*-Value < 0.05), resulting in: 69 proteins for the 6S + USA300-MRSA WT biofilm versus 6S + USA300-MRSA ΔMSCRAMM; 66 proteins for the 6S biofilm versus 6S + USA300-MRSA WT; 54 proteins for the 6S biofilm versus 6S + USA300-MRSA ΔMSCRAMM. Supplemental Data (upon request) show the detailed information of total proteins and unique peptide counts, as well as the annotation for each identified protein. Some proteins were quantified only in one condition, and in such cases, neither the p-values nor the fold changes (log2) could be computed. Nevertheless, proteins with a high intensity in one condition, but which were not present in the other condition, could have biological relevance, and a *q*-value (pseudo effect size and pseudo q.mod.) is provided so these proteins can integrate with proteins which have a fold change (see Experimental Section, Section 2.8).

When comparing 6S + USA300-MRSA WT to 6S + USA300-MRSA ΔMSCRAMM, most of the regulated proteins belonged to *V. dispar* (14 proteins), *S. oralis* (3 proteins), *S. mutans* (2 proteins), *F. nucleatum* (8 proteins), and. *S. aureus* (41 proteins). In 6S + USA300-MRSA WT, all the *V. dispar* proteins were upregulated: For *S. oralis*, glutamine synthetase (F5VX95) was found to be downregulated, while a carboxylase (F5VVU4) and a fructokinase (F5VTA2) were upregulated. Two *S. mutans* proteins were detected only in the 6S + USA300-MRSA ΔMSCRAMM samples: Q8DTD0, related to acyl-groups transferase, and Q8DUL2, glutamate dehydrogenase. As no uniquely or overlapped protein was characterized from *S. mutans* while in the 6S + USA300-MRSA WT, we assume that the overlapped protein, Q8DVD4, was probably downregulated in the 6S + USA300-MRSA ΔMSCRAMM. This protein is related to the *S. mutans* cell cycle and cell division. This result corroborates the reduced CFU counts for *S. mutans.*

The proteomic changes, which were promoted by the USA300-MRSA WT and the USA300-MRSA ΔMSCRAMM strain, clearly differed in multispecies oral biofilms, i.e., the MSCRAMM genes deletion appeared to compromise the regulatory impact on the 6S, based on the numbers of enhanced protein expression: comparing the 6S + USA300-MRSA WT with the 6S control biofilm, three proteins could be identified in the 6S control biofilm (1 for *V. dispar* and 2 for *F. nucleatum*), while 20 *S. aureus* proteins were exclusively identified in the 6S + USA300-MRSA WT biofilm. *S. oralis* in the presence of USA300-MRSA WT had a fructose-specific component upregulated (protein ID: F5VVS9). In addition, the regulated proteins originated from *S. aureus* were always upregulated in the 6S + USA300-MRSA WT biofilm, whereas the 6S + USA300-MRSA ΔMSCRAMM biofilm showed mostly downregulations.

**Figure 4:**
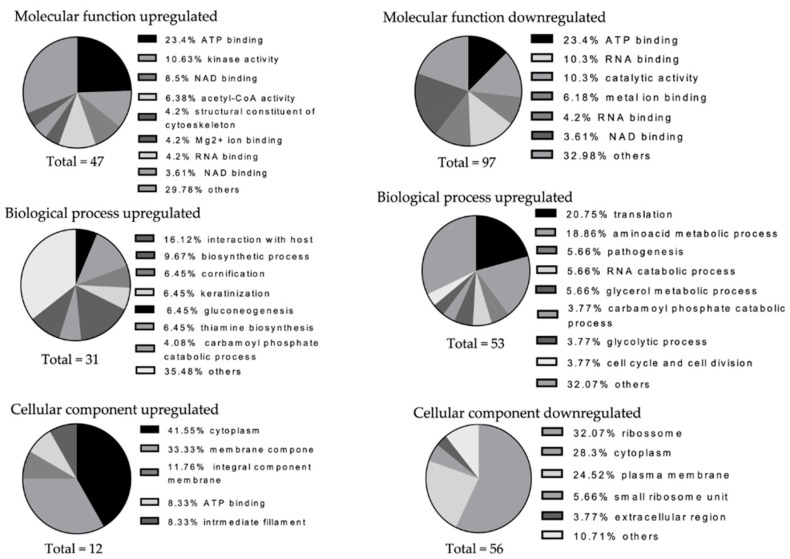
Annotation of regulated bacterial protein functions by gene ontology (GO) term enrichment. The comparison was performed between both *S. aureus* implementing biofilms (6S + USA300-MRSA WT versus the 6S + USA300-MRSA ΔMSCRAMM biofilm). The GO terms from all regulated proteins were categorized into three categories: molecular function, biological process, and cellular component, as displayed in the pie charts. The numbers of GO terms for each of the three categories are shown, whereas the proportion of each specific subcategory is also provided. Subcategories with GO terms less than 2% are classified as “others”. Total numbers are related to the total numbers of regulated proteins.

Taken that the USA300-MRSA WT and the USA300-MRSA ΔMSCRAMM strain induced different changes in the proteomic composition of the 6S control biofilm, we investigated functional differences of their regulated proteins. All individual proteins (including *S. aureus* proteins) were classified for their predicted molecular function, biological process, and cellular component (i.e., subcellular localization), based on their GO terms (Figure 4).

In brief, 47, 31, and 12 GO terms from the molecular function, biological process, and cellular component category, respectively, were generated from the proteins that were upregulated in the *S. aureus* biofilms, comparing the mutant in relation to the WT strain. The enriched GO terms from the downregulated proteins were in 97, 53, and 56 from these three domains. In general, both the up- and downregulated proteins in the *S. aureus* biofilms have diverse functions with 29.78 and 47.42% GO terms enriched in the “other” category in molecular function and 35.48 and 32.07% for the biological process domains. The downregulated proteins expressed at higher levels in the 6S + USA300-MRSA ΔMSCRAMM contained relatively large proportions of GO terms associated with the molecular function “RNA and ATP binding” (10.3 and 9.27%) and with the biological process “amino acid metabolic process” (18.86%). Most GO terms associated with the cellular components were “ribosome” (32.07%).

## 4. Discussion

Microorganisms naturally coexist either in the environment or in a host, adhering to surfaces or co-populating biofilms. This interaction varies greatly, ranging from coaggregation to antagonistic interactions. Interactions between *C. albicans* and *S. aureus*, for instance, are apparently synergistic or mutualistic and have been increasingly reported [41,42,43,44]. Of the ALS (agglutinin-like sequence) cell-wall proteins in Candida, Als3 (Q59L12) has been shown to regulate the adhesion of *C. albicans* to *S. aureus*, *S. epidermidis*, and *S. gordonii* [44,45,46,47,48]. In the presence of *S. aureus*, *C. albicans* expresses several proteins, such as a glyceraldehyde-3-phosphate dehydrogenase, identified as Q5ADM7. This was one of the positively regulated proteins in the 6S + USA300-MRSA WT biofilm versus the 6S + USA300-MRSA ΔMSCRAMM biofilm. The molecular role of this dehydrogenase involves the connection with the extracellular matrix and fibronectin. Aside from its molecular role, dehydrogenase biological process involves adhesion of the symbiont to the host and glucose metabolism. This result suggests that *S. aureus* might regulate the cell–cell communication between *C. albicans* and *S. aureus* via the fibronectin-binding proteins A and B (FnBPA and FnBPB–two of the encoding genes deleted in our mutant). Considering that both dehydrogenase and FnBPs have fibronectin as a ligand, this may represent an important network mechanism, not yet identified between *C. albicans* and *S. aureus* in a polymicrobial oral biofilm model.

Proteins on the cell surface have a pivotal role in interactions with microorganisms. Their external location facilitates a rapid function as communication and adherence elements. As cell-wall constituents, the proteins also function as a mechanical barrier and protect the cell from external stresses, while the cells can still perform division and morphogenesis [48]. *S. aureus* cell wall is composed of peptidoglycan and surface proteins, such as MSCRAMM, protein A, collagen-binding protein, and teichoic acids. Originally, MSCRAMM were defined as cell-surface-associated proteins, which interact with the host extracellular matrix [49]. Foster et al. (2019) [50], however, suggest limiting the term MSCRAMM to adhesions containing at least two IgG-like folded domains and that use the dock, lock and latch (DLL) ligand-binding mechanism. In this context, two MSCRAMM families can be further classified: MSCRAMM, which are related either to the clumping factor A (ClfA) of *S. aureus* or to SdrG of *S. epidermidis* (the Clf-Sdr-FnBP family). Secondly, there are MSCRAMM, which are similar to the collagen-binding protein of *S. aureus* (the Cna family), which can bind ligands by mechanisms, which involve larger conformational changes, e.g., ClfA and SdrG binding to fibrinogen via the DLL mechanism and Cna binding collagen by the collagen hug (CH). These bindings are rather strong, and a separation would require forces in the range of those which are necessary to break a covalent bond [51]. Hence, bacteria in moving fluids, such as in the bloodstream or in saliva, could be tied to these surfaces. Additionally, MSCRAMM, SdrC and FnBPs, facilitate cell aggregation and support the formation of biofilms [52,53,54,55].

A major innovation in our study was to use *S. aureus* strains containing markerless in-frame deletions of multiple MSCRAMM genes (for the clumping factor: ΔclfA and ΔclfB; for the fibronectin-binding proteins: ΔFnBPA-ΔFnBPB; for serine aspartate repeat proteins: ΔsdrC, ΔsdrD, and ΔsdrE). Therefore, most of the variability and cooperative binding-sites mechanisms between each MSCRAMM and common ligands could be avoided with these multi-locus mutants, rather than with an individual mutant. We report a significantly lower growth in USA300-MRSA ΔMSCRAMM in the 6S biofilm than in USA300-MRSA WT. Since MSCRAMMs mediate the initial attachment of *S. aureus* to host tissue, and this is a critical step in infection onset, the mutant strain is assumed to reduce the virulence of *S. aureus* WT in the supragingival biofilm model. Moreover, the qualitative analysis showed consistent results, since proteins were mostly downregulated in the ΔMSCRAMM-containing biofilm. Thus, our data suggest that the deletion of MSCRAMM genes may compromise microorganism viability and cell replication in a biofilm. This may also explain why the classical *S. aureus* agglutination as a bunch of grapes was rarely seen in the CLSM images for 6S + USA300-MRSA ΔMSCRAMM. The creation of a dysbiotic polymicrobial biofilm depends on the ability of primary colonizers to attach and colonize surfaces. In oral diseases, these primary colonizers are often *Streptococcus* and *Actinomyces* species that specifically adhere to salivary proteins bound to the tooth surface or to epithelial cells [56,57]. *S. gordonii* is one of these early enamel tissue colonizers and binds to the glycoproteins present in saliva via its streptococcal surface proteins (SspA and SspB), providing new adhesion sites for secondary colonizers, such as *P. gingivalis* and *F. nucleatum*. An analysis of the supragingival phenotype in the presence of *S. aureus* generally showed higher *S. mutans* and *S. oralis* CFU counts. This might not represent urgent shifts into an oral disease but does represent a dysbiotic biofilm that can create more viable sites for secondary colonizers and further. This mutualism builds a protective structure against host response, and consequently leads to increased chances in disease pathogenesis. Considering that USA300-MRSA ΔMSCRAMM in the 6S biofilm downregulated proteins in *S. mutans*, reduced CFU counts, and changed its distribution within the supragingival biofilm, *S. aureus* and both *S. mutans* and *S. oralis* must have developed cell–cell communication. CLSM images showed that USA300-MRSA cells were more abundant, co-located with *S. mutans* and *S. oralis*, forming an aggregated structure grouped inside the biofilm. This result corroborates previous knowledge about the importance of MSCRAMMs in *S. aureus* attachment and further highlights the importance of MSCRAMMs for *S. aureus* in a multispecies biofilm containing streptococci. *S. aureus* can be a commensal pathogen found in the oral cavity of healthy patients, inside periodontal pockets, within an hour after surgically inserting titanium dental implants [58]. Although the transient status of oral *S. aureus* may not cause diseases, our findings showed that *S. aureus* caused dysbiosis. Wild *S. aureus* co-aggregated into the oral microbiota, especially with primary enamel colonizers such as streptococci, shifted the numbers of secondary colonizers responsible for the structuring of biofilm architecture and ensured the first step towards disease development.

On the other hand, as they play a wide array of roles in infection and host colonization, the importance of surface proteins such as *S. aureus* MSCRAMMs becomes increasingly more relevant as immunotherapeutic targets. *S. aureus* MSCRAMMs clearly affect *S. aureus* virulence in in vitro supragingival biofilm model. This means *S. aureus* MSCRAMMs seem to be an important CWA not only for *S. aureus*, but also in the communication between streptococcal cells. Interestingly, there is homology of LPTX adhesions within this species, with fibronectin as a common ligand. Corroborating this knowledge, downregulated *S. mutans* proteins in a six-species biofilm with additional ΔMSCRAMM were mostly related to amino acid synthesis and the binding of metal ions such as Mg2+. These metal ions work as co-factors that enhance the role of SrtA, which is responsible for recognizing the LPXTG motif and using it to anchor the protein that shall carry it to a peptidoglycan cell-wall building block [59]. The CLSM images show that a physical proximity between *S. aureus* and streptococci was interrupted by the mutant strain, as opposed to the USA300-MRSAWT effect. Overall, cell-to-cell communication via MSCRAMM is a keystone for USA300-MRSA interaction and development in a *Streptococcus*-containing biofilm.

Collagen-binding proteins (CBPs) are well known for mediating a *Streptococcus* specific binding to collagenous host tissues, often resulting in deep tissue and persistent infections [60]. Two surface proteins with CBPs properties were identified in UA159, the first sequenced *S. mutans* strain in UA159: SpaP surface adhesin and cell-wall-associated protein A (WapA). Protein A is an LPXTG cell-wall-anchored protein [61,62]. WapA, initially called antigen A, was considered a promising vaccine candidate for tooth caries based on immunization studies conducted with rodents, monkeys, and human volunteers [63]. Although vaccination efforts against tooth caries using WapA have not been successful [64], these studies have proven that *S. mutans* SpaP and WapA proteins bind collagen in vitro. More recently, molecular and genomic analyses of *S. mutans* isolates have led to the identification of two additional CBPs, Cnm and Cbm [65,66,67,68,69]. Clinical and laboratory studies with Cnm+ and Cbm+ isolates have strongly suggested that these CBPs are important virulence factors in systemic infections [70,71,72,73,74], and more recently, in caries severity [75]. Interestingly, there is a fibronectin-binding protein in *S. pyogenes,* identified with protein ID: A0A0H2UTI7, which has 100% homology with *S. mutans* CBPs; therefore, they are likely to have fibronectin as a common ligand with *S. aureus* FnBPB. An investigation of fibronectin-binding protein alone in both *S. aureus* and *S. mutans* as potential targets to reduce *Streptococcus* adherence in the supragingival biofilm model, and their role as early colonizers to prevent dysbiotic biofilm from shifting into infections such as caries and periodontal diseases seems very promising.

When MSSA isolates were added to the 6S biofilm (PN35 and OMI1122), *V. dispar* CFU counts did not increase compared to the addition of MRSA (HU13N and SF8300). Furthermore, a glucosyltransferase from *V. dispar* was only quantified with highly normalized intensity when SF8300 was added to the supragingival biofilm. Intriguingly, *S. mutans*-secreted glucosyltransferase also caused binding to the Candida surface and synthesized glucans in situ. Glucans formed on the surface, enhancing bacterial–fungal co-adhesion and embedding microorganisms in an EPS-rich matrix, promoting mixed-biofilm accumulation [76]. Biofilms are known to be produced by distinct mechanisms in MRSA and MSSA. Supplementing certain chemicals to growth media affects biofilm formation of *S. aureus* strains by regulating gene expressions or breaking biofilm-forming bonds. For example, growth media pH decreased due to glucose degradation, as described for the *S. mutans* mechanism during caries development, repressing the agr regulator system of *S. aureus* strains. Thus, growth media supplemented with glucose represses agr regulator systems, which increases biofilm formation [77]. In fact, metabolic communication in the matrix and quorum sensing detection between microorganisms through small-secreted signalling molecules are essential factors in understanding how cells communicate [78] or interact during biofilm formation [79]. The exchange of electrons might be particularly intriguing as ion-binding protein expression was the fourth quantified downregulated protein in the presence of an MSCRAMM mutant. In the supragingival multispecies *S. aureus* biofilm, surface adhesins could have allowed microorganisms to obtain energy from different reactions. The production of phenazine in *P. aeruginosa* and ethanol in *C. albicans* during biofilm formation have been described as positive feedback loop, which seems relevant to human health. The formation of *P. aeruginosa* biofilms in bronchial epithelial cells was thereby increased, such as the virulence of *P. aeruginosa* and *C. albicans* in the host [79].

The MSSA biofilm formation is ica-dependent (PIA-dependent, encoded by an icaADBC gene), whereas the MRSA biofilm formation is ica-independent (PIA-independent, by surface proteins containing LPXTG anchoring domain). The agr quorum-sensing leads to a reduced biofilm formation, which is based on the positive regulation of phenol-soluble modulins (PSM), such as surfactants, proteases, and nucleases. These PSM degrade the biofilm matrix and thereby disperse microorganisms in the biofilms enzymatically [80,81]. It was shown that MRSA mutants with icaADBC operon-deletion were not impaired in biofilm formation [21], whereas icaADBC operon-deleted MSSA mutants were affected during biofilm development. We analyzed the difference between MRSA and MSSA in the in vitro supragingival biofilm model and compared USA800-MRSA, HU13N, a nasal MRSA isolate, with ST72-MSSA, PN35, an MSSA isolated from the periodontal pocket. Not surprisingly, the MSSA strain had lower growth numbers in the biofilm than the USA800-MRSA HU13N. In addition, the total biofilm CFU counts showed a tendency towards decreasing numbers in both MSSA strains tested, ST72-MSSA PN35 and ST72-MSSA OMI1122, compared to the MRSA strains (USA800-MRSA HU13N and USA300-MRSA SF8300). *Staphylococcus* strains, which are methicillin-resistant, possess a *mec* operon, whereas methicillin-sensitive *Staphylococcus* strains lack this operon [82]. Studies investigating the association between methicillin resistance and biofilm phenotype revealed a decrease in biofilm formation by HA-MRSA, if the SCC*mec* was removed, inducing an upregulation of the protease activity [83,84]. The biofilm formation of MRSA was enhanced due to the *psm-mec*-encoded phenol-soluble modulin mec (PSMmec) and the *mecA*–encoded penicillin-binding protein 2a (PBP2a). Both proteins impair the MRSA virulence. Similar to other *S. aureus* virulence toxins, the expression of PSMmec is regulated by an agr two-component signal transport system [82,85]. These molecular mechanisms induce the dispersal of some cells after *S. aureus* biofilm initiation and retain small foci of biofilm growth [8]. These foci can then further mature into the so-called biofilm tower structures. The “exodus”-termed early dispersal phase requires the *sae* system and the *sae*-regulated nuclease, while it is independent of the agr system. The virulence response systems are triggered if the auto inducer peptide *of S. aureus* exceeds a certain threshold, which is the case if the bacterial population increases. This induces the production of virulence factors and antibiotic resistance mechanisms, such as the upregulation of the efflux pump. Hence, the inhibition of QS could avoid the killing of host cells by *S. aureus*, as well as its evasion of the host immune response and its effective dissemination. These pathogenic abilities of *S. aureus* are based on the production of QS-regulated toxins, such as delta-toxin, Panton–Valentine leukocidin, and the staphylococcal enterotoxin C [86]. Our results determined a clear difference between MRSA and MSSA CFU counts inside the supragingival biofilm model, as well as a lower number of species-specific taxonomies, e.g., *C. albicans* and *S. mutans*, when MSSA strains were added to the six-species biofilm. Although the existence of different biofilm phenotypes between MRSA and MSSA are also associated with virulence factor, our supragingival model does not allow for stating that the presence of LUK PVL gene was solely responsible for this variability.

## 5. Conclusions

The present study underlined the importance of MSCRAMM in an established and characterized supragingival biofilm model grown on hydroxyapatite disks by individually integrating *S. aureus* isolates and the multilocus mutant for MSCRAMM. The co-presence of components in the oral environment, such as proteins/cytokines in saliva, transient microbials, or biomaterials, may contribute to the disease pathogenesis and its multifactorial aetiology. In a synergistic interaction of microbial communities with a transient microorganism (i.e., *S. aureus*), either an adequate niche between the two is generated or products are secreted by one species that serve as nutrients for the other. The result of this seems to trigger the change from a homeostatic biofilm to a dysbiotic biofilm towards the development of diseases, in addition to the inefficiency of the immune system.

## Figures and Tables

**Table 1:** *S. aureus* strains used in this study and the respective terminology throughout the manuscript.

S. aureus [Terminology]	Place of Isolation, Description	Relevant Characteristics	PVL	Source
SF8300_USA300 wild type [USA300-MRSA WT]	San Francisco, California, USA, CA-MRSA from an abscess	Multi-resistant ^1^ USA300, CC8, SCCmec IV ^2^	+	[28]
HU13N [USA800-MRSA]	Rio de Janeiro, Brazil, CA-MRSA nasal isolate	Non-multi-resistant ^3^, USA800, CC5, SCCmec IV ^2^	−	[29] This study
PN35 [ST72-MSSA-]	Rio de Janeiro, Brazil, MSSA, periodontal pocket isolate	Methicillin-sensitive ^4^, ST72/CC8-	−	This study
OMZ1122 [ST72-MSSA+]	Seattle, USA, ATCC 25923	Methicillin-sensitive, ST72	+	[30]
SF8300_ΔMSCRAMM [USA300-MRSA ΔMSCRAMM]	Mutant strain from SF8300_USA300	*In-frame* deletions of *clfA*, *clfB*, *sdrC*, *sdrD*, *sdrE*, *fnbA-fnbB*	+	This study

+, positive; −, negative; CA, community acquired; ^1^ methicillin-resistant *S. aureus* (MRSA): resistance to mupirocin, clindamycin, tetracycline, methicillin and beta-lactams; ^2^ staphylococcal cassette chromosome mec (SSCmec); ^3^ methicillin-sensitive *S. aureus* (MSSA): resistance to erythromycin, ciprofloxacin, methicillin and beta-lactams; ^4^ resistance to erythromycin and benzylpenicillin; PVL = leukocidin Panton–Valentine; *clfA* and *cflB*, correspond to clumping factor A and B; *sdrC*, *sdrD* and *sdrE*, correspond to serine aspartate-repeat protein C, D and E; *fnbA* and *fnbB*, to fibronectin-binding protein A and B.

**Table 2:** Sequence and formamide concentrations of FISH probes used in this study.

Organism	Name	FA ^1^ (%)	WB ^2^ (mM)	Sequence (5′, 3′)	Source
*S. aureus*	Saur229	40	46	CTAATGCAGCGCGGATCC	[7]
*S. oralis*	MIT446	25	149	ACACYCGTTCTTCTCTTACAA	[10]
*S. mutans*	MUT590	30	112	ACTCCAGACTTTCCTGAC	[36]

^1^ Formamide concentration in the hybridization buffer; ^2^ Concentration of NaCl used in the washing.

## Data Availability

Data is contained within the article or supplementary material. The data presented in this study are available in the Supplemental Data, Tables S1 and S2.

## Supplementary Materials

The following are available online at https://www.mdpi.com/2079-6382/10/2/116/s1, Supplemental Data: Protein identification and quantification results, including normalization data and visualization plots. Table S1: Detailed information of the genotype, virulence gene profile, and antibiogram of PN35 and HU13N clinical isolates. Table S2: Primers used for the construction of in-frame deletions of clfA and clfB, for the construction of triple deletion of *sdrC-sdrD-sdrE* genes and double deletions of *fnbA* and *fnbB* genes.

## Abbreviations

FISH	Fluorescent in situ hybridization
CLSM	Confocal laser scanning microscopy
PIA	Extracellular polysaccharide intercellular adhesion
CWA	Cell-wall-anchored proteins
MSCRAMM	Microbial surface that recognizes adhesive matrix molecules
clf	Clumping factors
sdr	Serine aspartate repeats proteins
fnBP	Fibronectin-binding proteins
MRSA	Methicillin-resistant *S. aureus*
MSSA	Methicillin-sensitive *S. aureus*
icaADBC	Operon-encoded polysaccharide intercellular adhesin
eDNA	Extracellular DNA
MLST	Multilocus sequence type
SCC	Staphylococcal cassette chromosome
PVL	Panton-Valentine leukocidin (PVL)-encoding genes (*lukF-PV* and *lukS-PV*)
CBA	Columbia blood agar
TSB	Tryptic soy broth
SSmec	Staphylococcal cassette chromosome mec
mFUM	Fluid universal medium
CFU	culture forming units
HA	Hydroxyapatite disk
LC-MS	Liquid Chromatography-Mass Spectrometry
HIFU	High Intensity Focused Ultrasound
DDA	Data-dependent analysis
LIMS	Laboratory information management system
FDR	False discovery rates
GO	Gene ontology
ALS	Agglutinin-like sequence
DLL	Dock, lock and latch mechanism
CH	Collagen hug
Ssp	Streptococcal surface proteins
CBPs	Collagen-binding proteins
PBP2a	penicillin-binding protein 2a
