# Supplementary material for: Staphylococcus aureus Interferes with Streptococci Spatial Distribution and with Protein Expression of Species within a Polymicrobial Oral Biofilm
Source: Antibiotics (Basel). 2021 Jan 26;10(2):116. doi: 10.3390/antibiotics10020116 (PMC7911025; doi:10.3390/antibiotics10020116)
Supplement: Supplementary file 1 [file antibiotics-10-00116-s001.zip › Supplements/Supplemental data 1_Proteomics/╬öMSCRAMMs biofilm vs control biofilm/MQ-report_╬öMSCRAMMS vs control biofilm.pdf]

FGCZ Two-Group Analysis: MQ-report-p2687-workunit_165007_Rep1+2+3_MSCRAMMS_Ctrl Statistics for a Quantitative Protein Matrix

Functional Genomics Center Zurich

19 June, 2018

Contents

1 Input Matrix **1**
1.1 The Numbers . . . 1
1.2 The Groupings . . . 2
1.3 The Protein Quantities . . . 3

2 Quality Control **5**
2.1 Distribution of Intensities . . . 5
2.2 Normalization . . . 5
2.3 Coefficients of Variations . . . 7
2.4 Heatmaps and Clustering for Samples and Proteins . . . 11

3 Two Group Analysis **14**
3.1 Adjusted moderated p-values (q-values) (limma output) . . . 15
3.2 Proteins Quantified in only one condition . . . 17
3.3 List of Columns in the output table . . . 21

4 References **21**
4.1 Disclaimer . . . 21

5 Session Info **22**

1 Input Matrix

Experiment is called: MQ-report-p2687-workunit_165007_Rep1+2+3_MSCRAMMS_Ctrl

1.1 The Numbers

The protein matrix is filtered like this:

- Minimum number of peptides / protein: 2

- Maximum of missing values per protein : 4
- The total number of proteins in this experiment is: 1557
- Total number without decoys sequences is 1557
- Percentage of contaminants : 0.8 %
- Percentage of false postivies : 0 %

1.2 The Groupings

Condition	# samples
Group1	3
Group2	3

Here the files in each group:

Condition	Raw.file
Group1	06_6_species_1
Group1	10_6_species_2
Group1	15_6_species_3
Group2	08_6_sp_MSCRAMMs_3
Group2	09_6_sp_MSCRAMMs_2
Group2	14_6_sp_MSCRAMMs_1

	name
reference	Group1
condition	Group2

1.3 The Protein Quantities

The input matrix has the following structure (Figure 1).

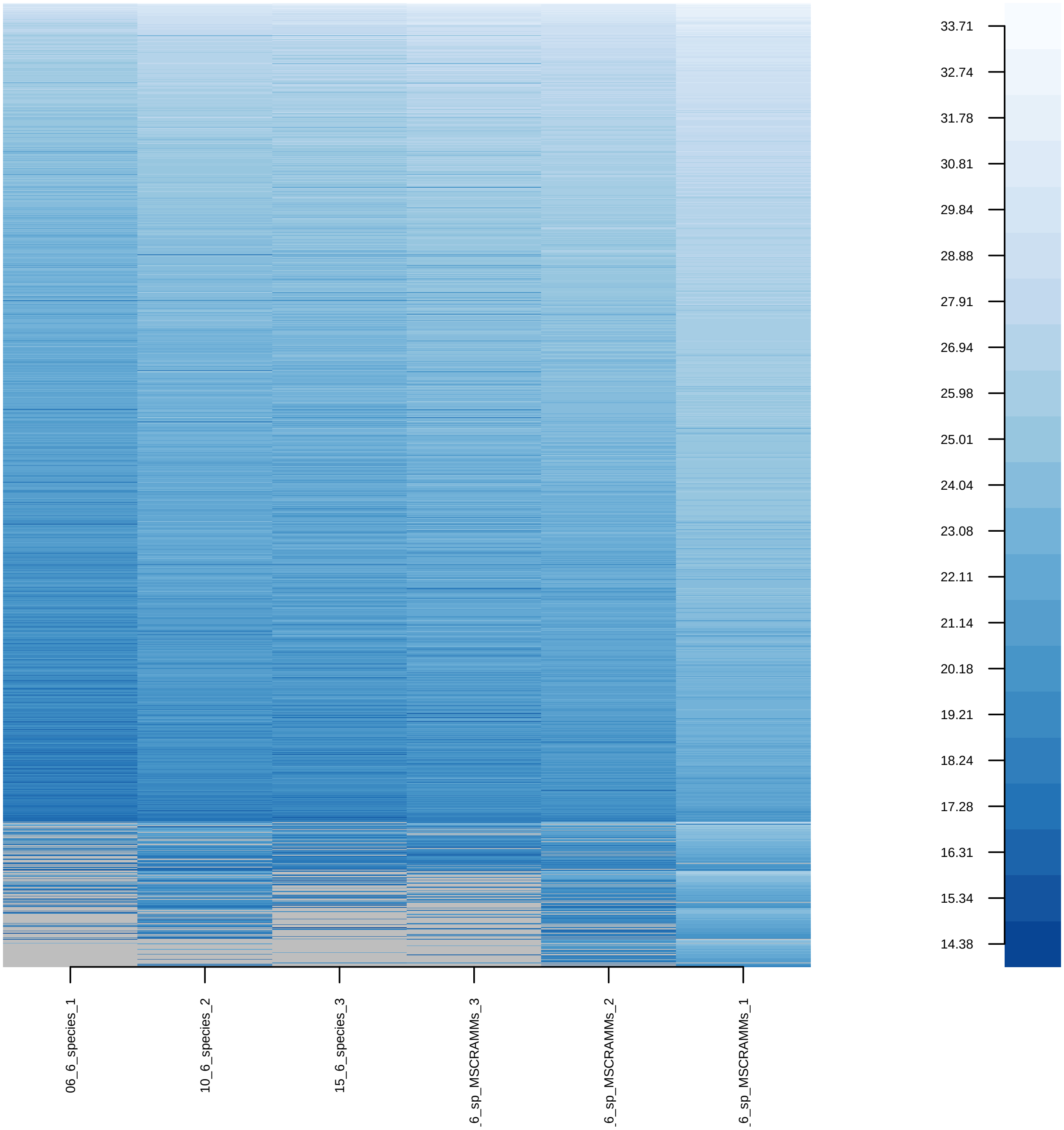

Figure 1: Heatmap for quantifyable proteins sorted by missigness and intensity (log2). Gray - missing values

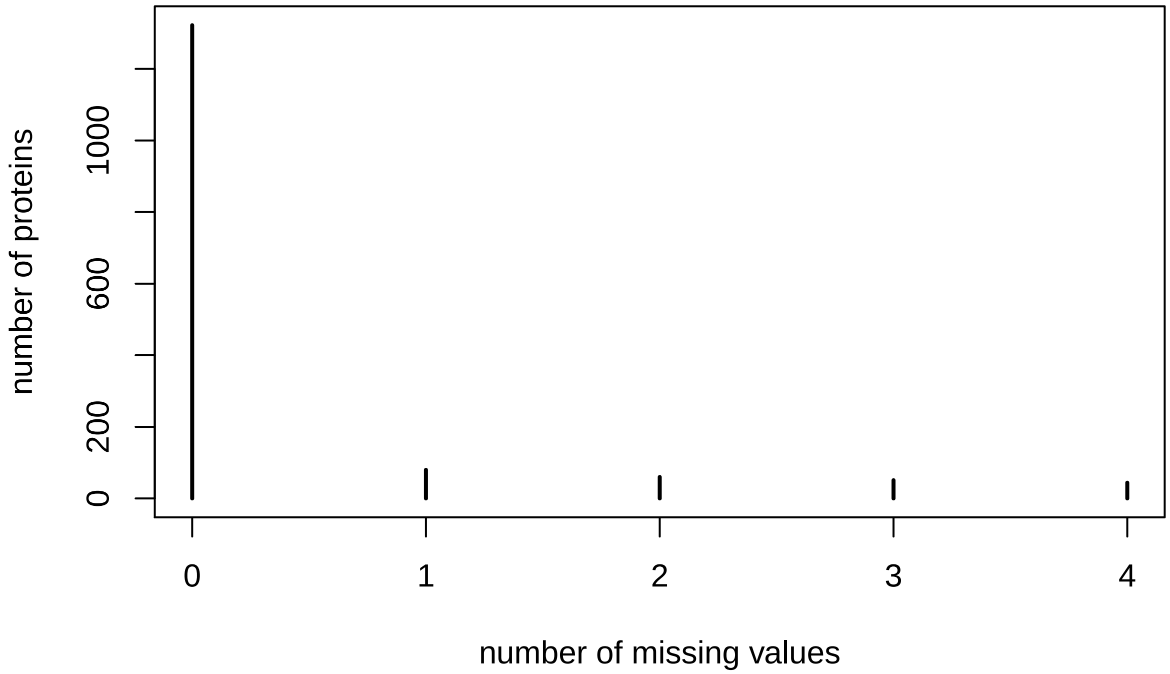

Figure 2: # of missing values per protein

2 Quality Control

2.1 Distribution of Intensities

Shown in Figure 3 are the distributions of raw log2 transformed intensity values. Ideally the violins should look very similar.

In Figure 4 the log2 fold change of the average sample intensity versus the mean average intensity of all samples is shown. It is critical if a samples average deviates more than 5 times from the average of all samples.

2.2 Normalization

Figure 5 shows the normalized values. Normalization is applied to remove systematic differences in protein abundance due to different sample concentrations, so that differentially expressed proteins can be detected. To this task the z-score of the log2 transformed intensities is computed, which in addition is multiplied by the average of the standard deviation of the log2 transformed intensities in all samples. After normalization all samples have a similar distribution.

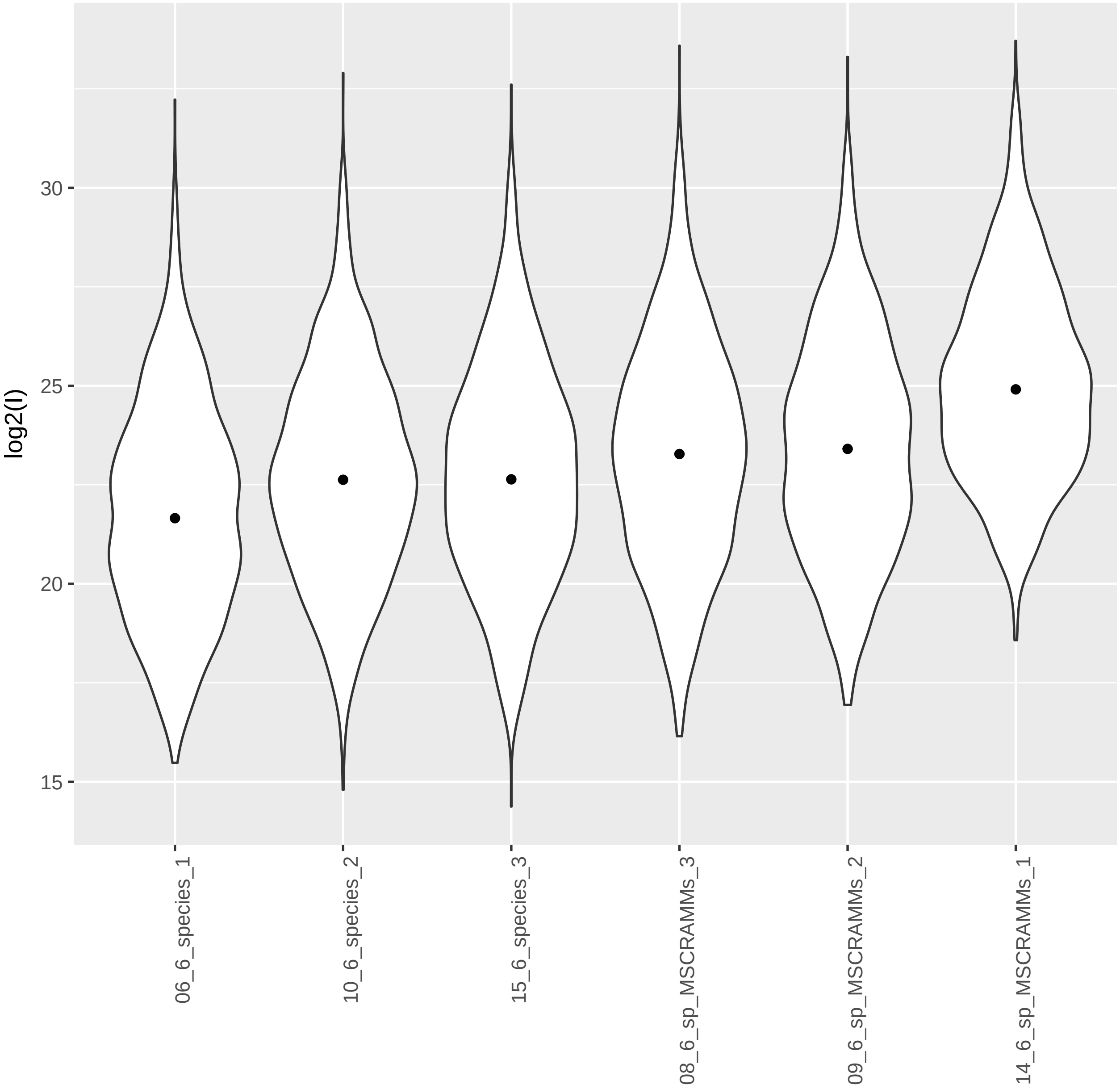

Figure 3: Density plot for quantifyable proteins (log2 transformed)

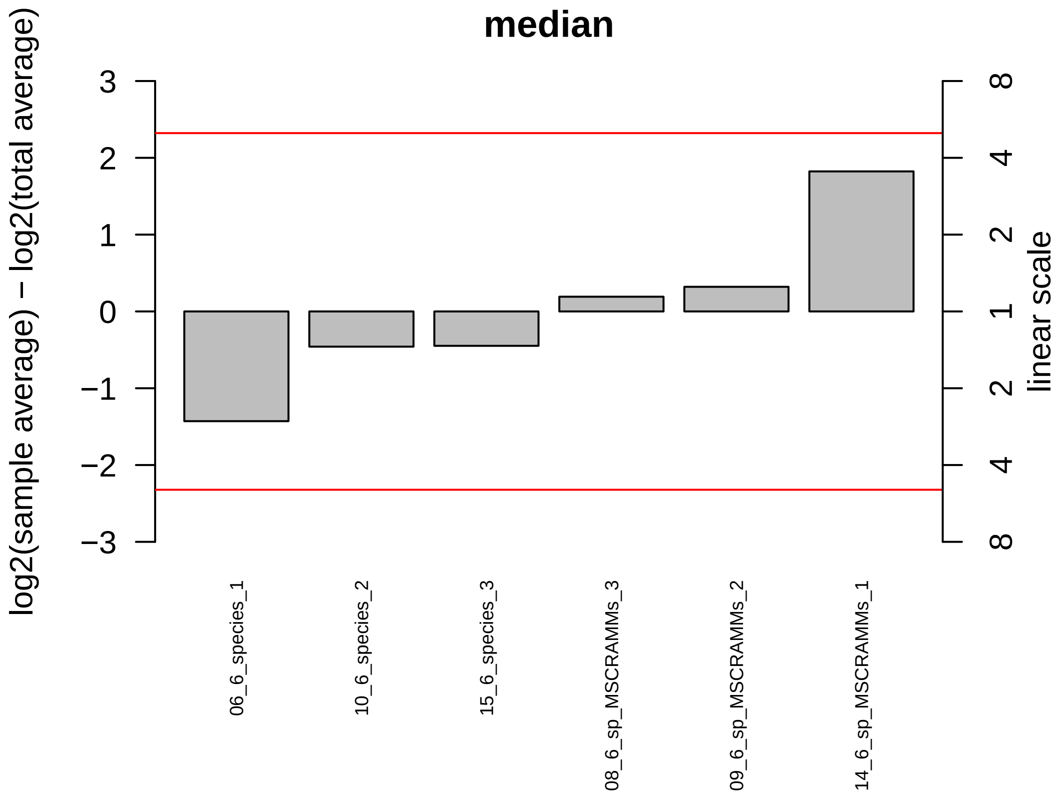

Figure 4: Average intensity in sample vs average intensity in all samples. red line - critical fold change.

Table 1: median of cv

condition	cv
Group1	44.60469
Group2	73.67988
all	95.33392

2.3 Coefficients of Variations

The Figure 6 show the coefficient of variations for all proteins in each condition and overall. To observe differences between conditions the variation within a condition should be smaller than within all conditions.

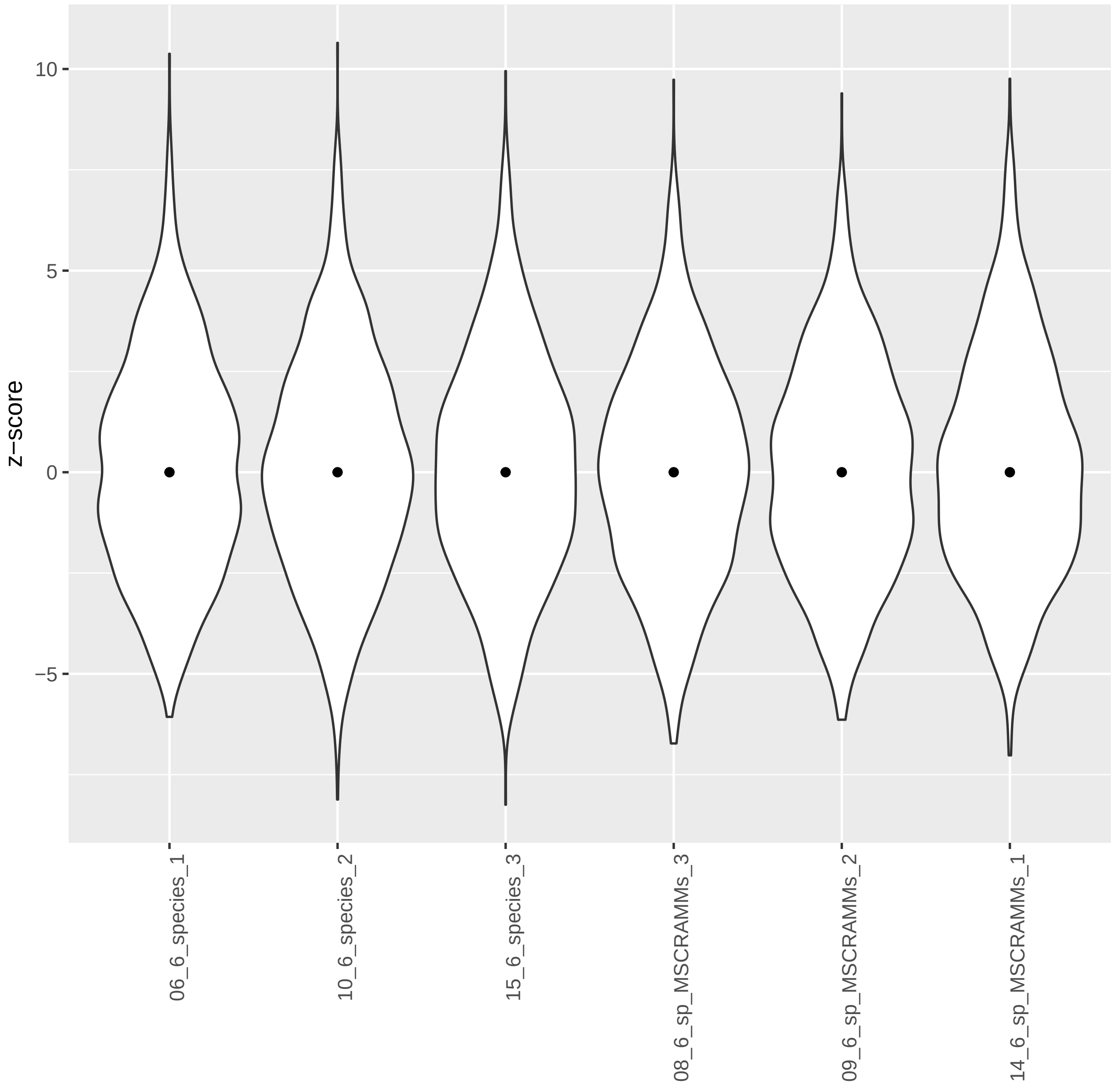

Figure 5: Density plot for normalized values (z-score)

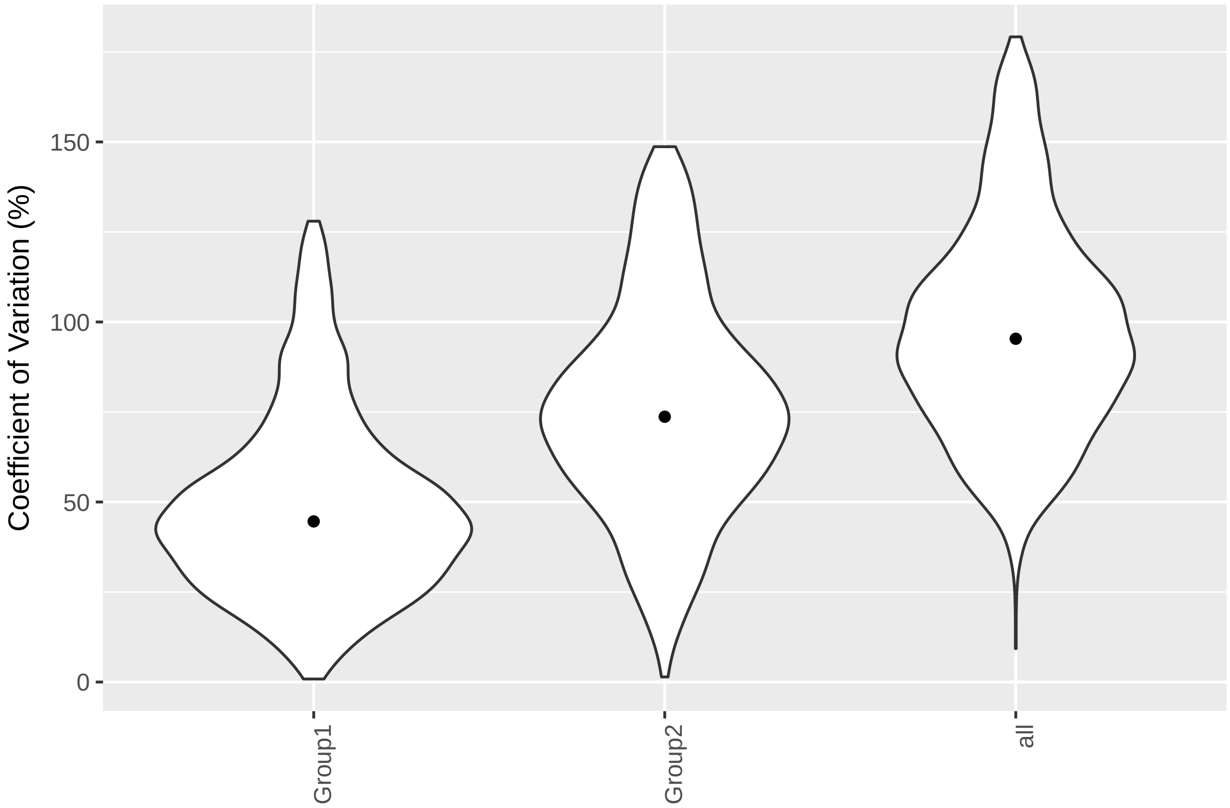

Figure 6: Distribution of protein CV within condition and overall

Table 2: median of sd

condition	sd
Group1	0.4467671
Group2	0.4870792
all	0.5267082

The Figure 7 shows the distributions of standard deviations for all proteins with the conditions and overall after transforming and normalization the data. To observe differences between conditions the standard deviation within a condition should be smaller than within all conditions.

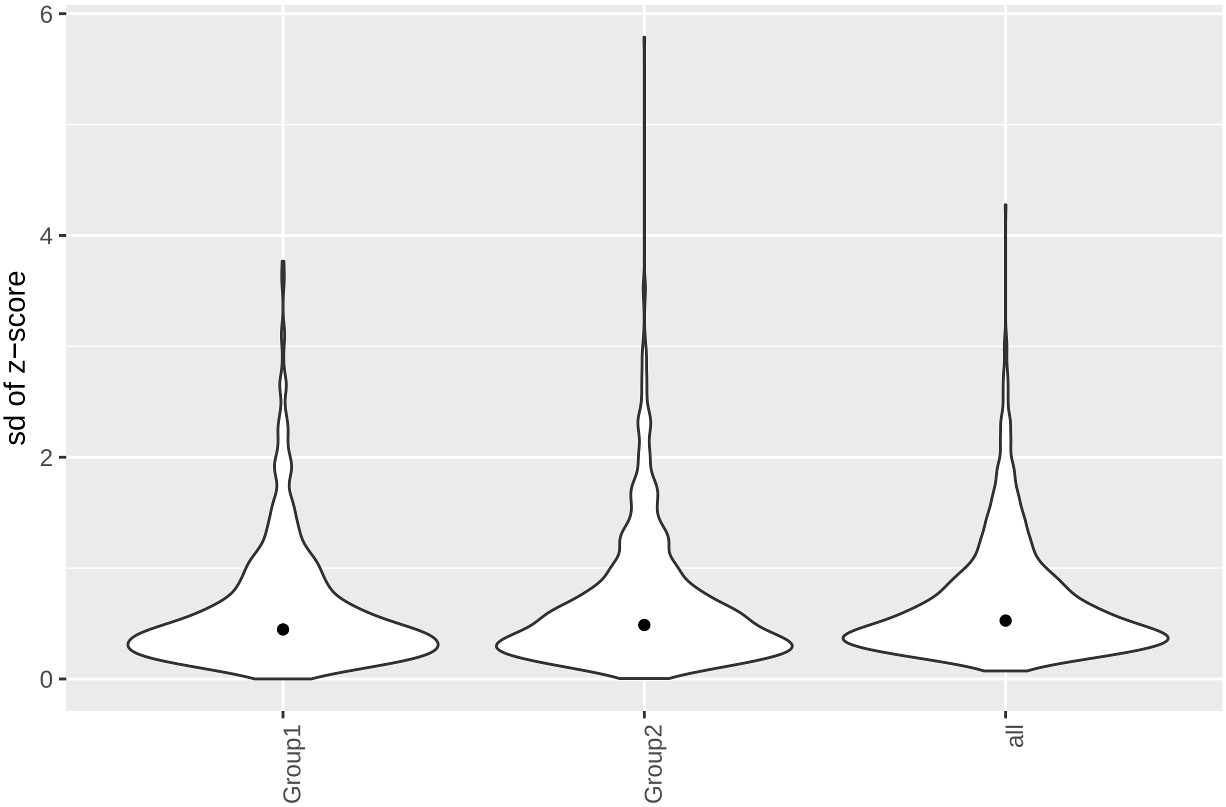

Figure 7: Distribution of protein standard deviation (after sample normalization and scaling) within conditions and overall

2.4 Heatmaps and Clustering for Samples and Proteins

In Figure 8 and Figure 9 we show how samples are clustering depending on their correlation and on the protein expression profiles.

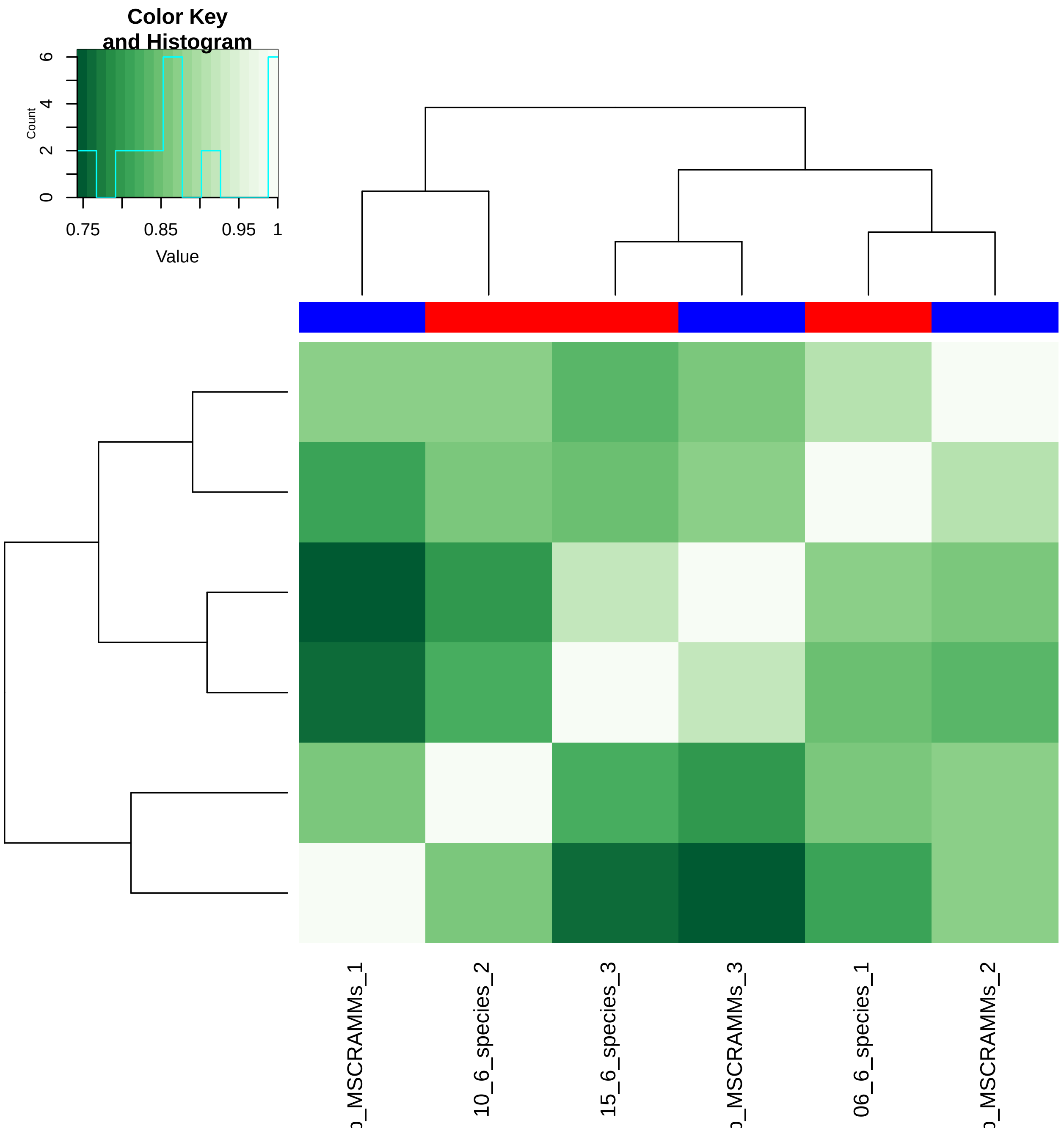

Figure 8: Heatmap of correlations (spearman) between samples.

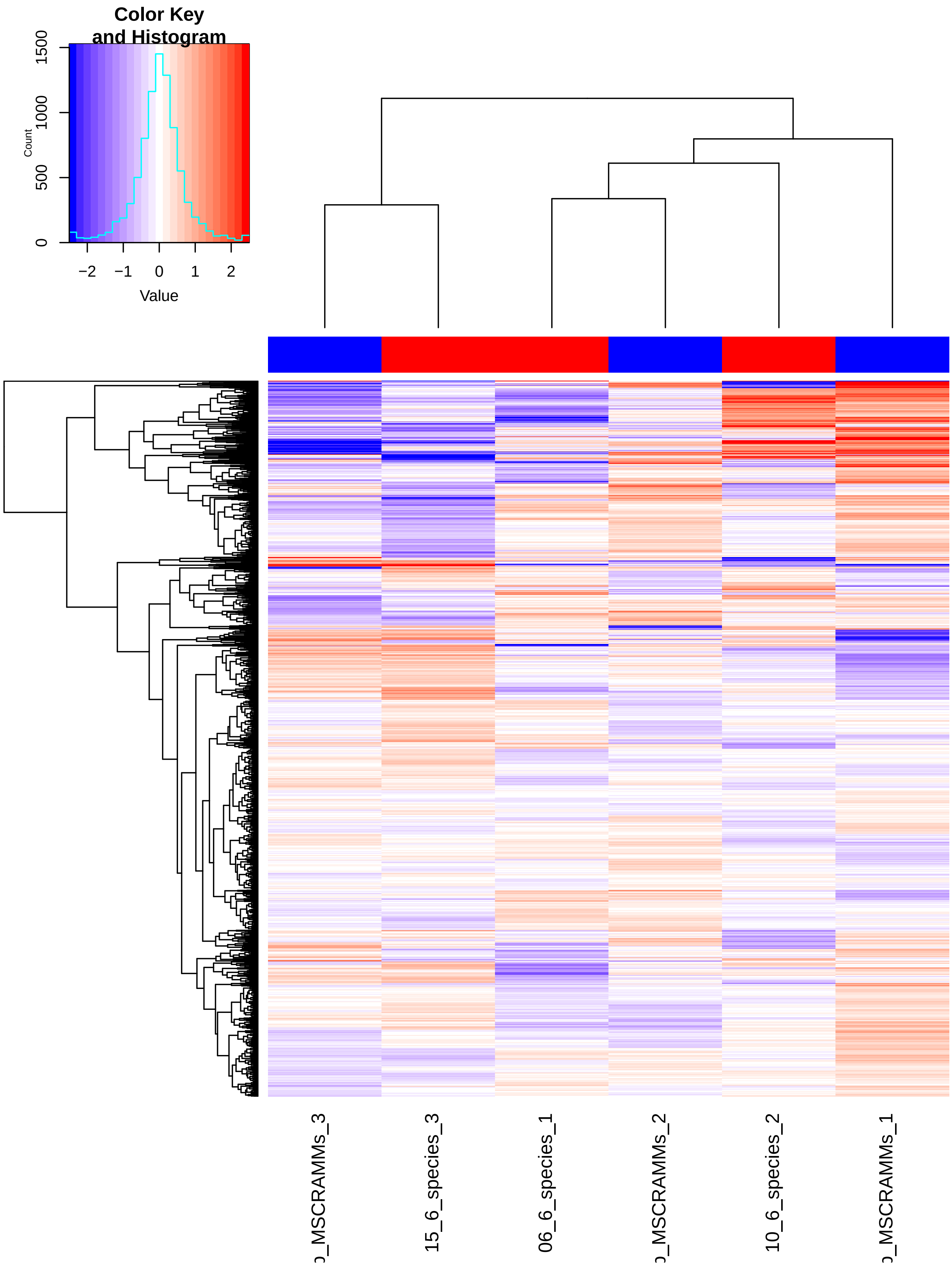

Figure 9: Heatmap of normalized data.

3 Two Group Analysis

In the following analysis we assume that most of the proteins are not regulated (fold change is equal zero). p-values and Q-values are a measure of how likely it is to observe the data given the assumption that they are not differentially regulated. Small p-values tell us that H_0 (no regulation) is very unlikely. Figure 10 shows the distribution of fold changes. Most of the fold changes should be close to zero and also the median of all fold changes (red dashed line) should be close to zero (green line).

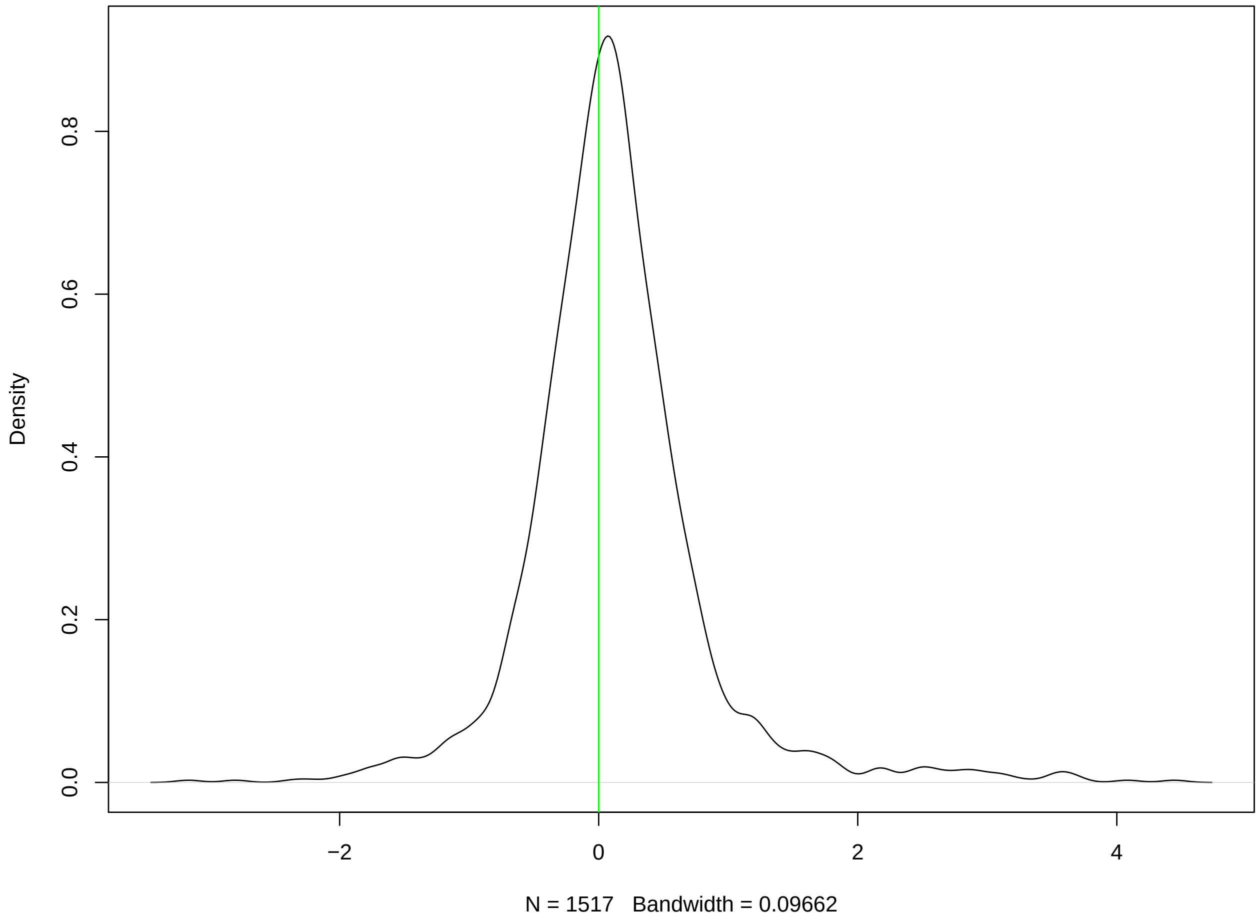

Figure 10: Distribution of log2(FC). red dashed line - median fold change.

3.1 Adjusted moderated p-values (q-values) (limma output)

If the groups are different we would expect more small p-values in Figure 11 than by chance (blue horizontal line). If there are only as many or less small p-values as by chance than no significant false discovery rate controlled calls (Q Values) will be made in Figure 12.

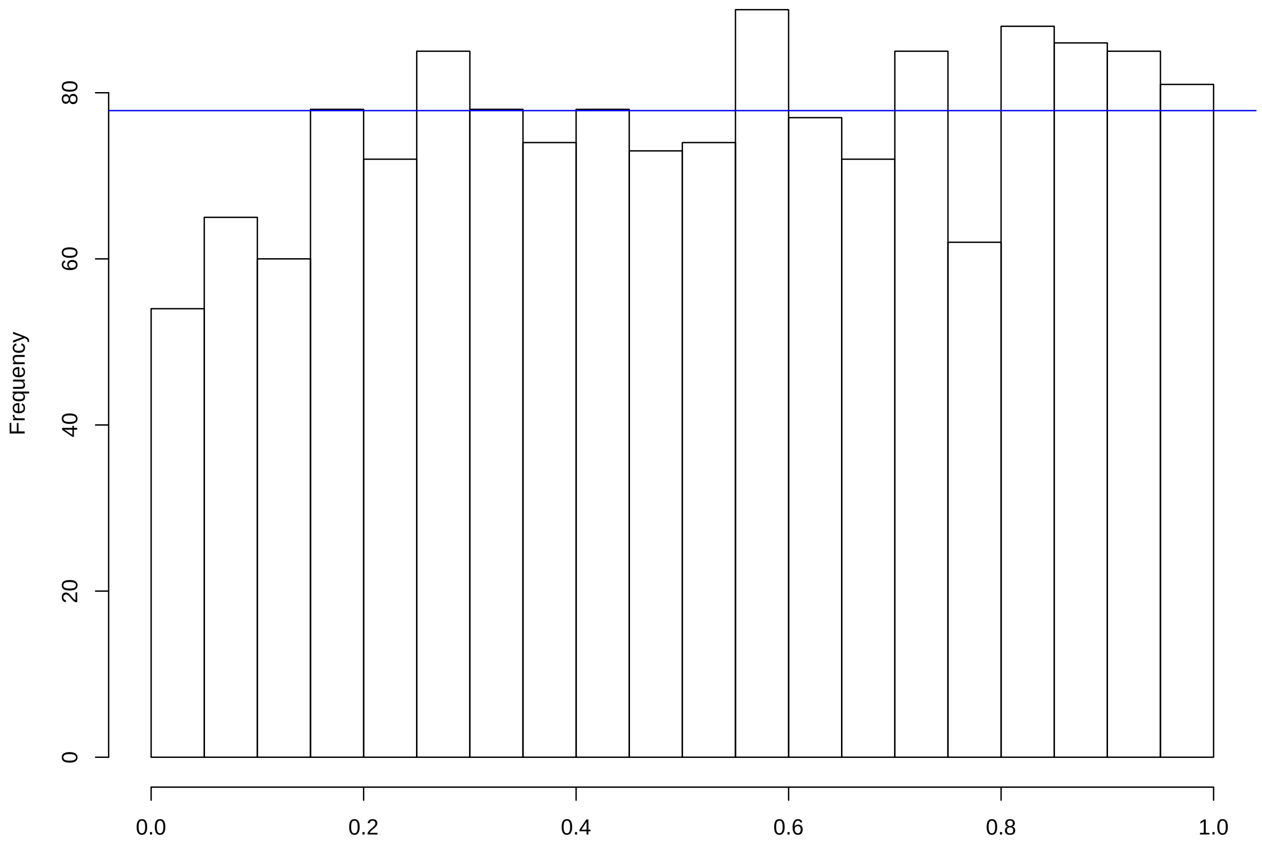

Figure 11: Histogram of moderated p values.

Significant calls are made with q-value smaller than 0.1 (false discovery rate, computed with bioconductor package qvalue) (see Figure 12)

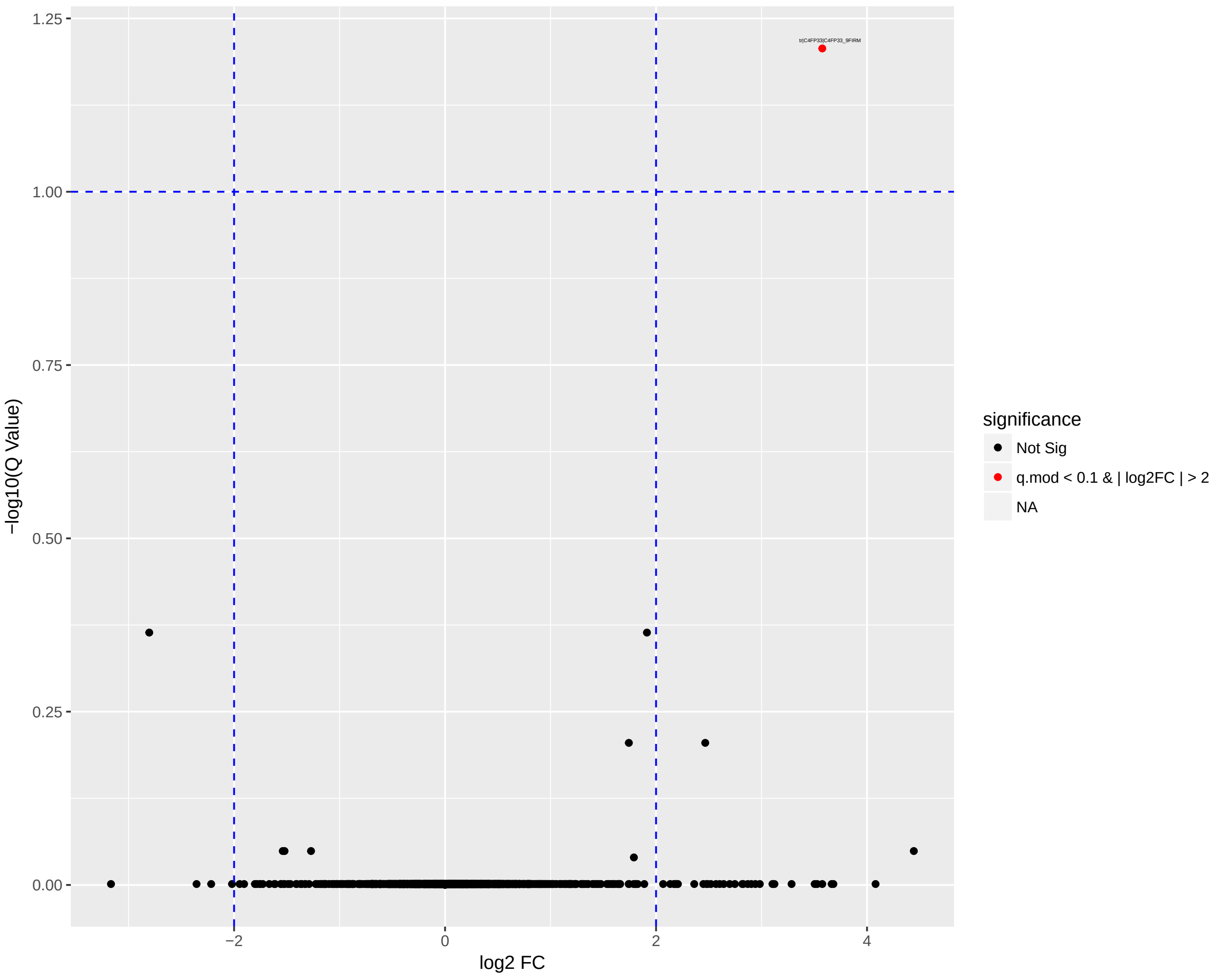

Figure 12: VolcanoPlot : x axis log2 fold change of normalized data, y axis -log10(Q-value).

Table 3: Number of not significant and significant proteins.

Var1	Freq
Not Significant	1516
Significant	1

Table 4: Top 20 proteins sorted by smallest Q Value (q.mod). The effectSize column is the log2 FC of condition - reference.

TopProteinName	effectSize	q.mod
tr|C4FP33|C4FP33_9FIRM	3.5759027	0.0621267
tr|C4FN74|C4FN74_9FIRM	1.9136688	0.4324286
tr|Q7P350|Q7P350_FUSNV	-2.8043590	0.4324286
tr|C4FQV9|C4FQV9_9FIRM	2.4664272	0.6237431
tr|F9EMJ0|F9EMJ0_FUSNU	1.7421524	0.6237431
tr|A0A0H2XIK4|A0A0H2XIK4_STAA3	4.4435996	0.8934278
tr|C4FNL7|C4FNL7_9FIRM	-1.2706413	0.8934278
tr|Q7P791|Q7P791_FUSNV	-1.5220480	0.8934278
zz|FGCZCont0260_FGCZCont0260|FGCZCont0260	-1.5385574	0.8934278
tr|C4FQA3|C4FQA3_9FIRM	1.7898974	0.9125880
CON___P02662	1.2949143	0.9968787
sp|O06942|DNAK_STRMU	-1.1378265	0.9968787
sp|O07329|CCPA_STRMU	-1.6660591	0.9968787
sp|P01591|IGJ_HUMAN	0.7915166	0.9968787
sp|P01833|PIGR_HUMAN	-0.5184913	0.9968787
sp|P01834|IGKC_HUMAN	0.5363838	0.9968787
sp|P01857|IGHG1_HUMAN	0.6740326	0.9968787
sp|P01871|IGHM_HUMAN	0.7974068	0.9968787
sp|P01876|IGHA1_HUMAN	0.1584700	0.9968787
sp|P04264|K2C1_HUMAN	-0.0464545	0.9968787

3.2 Proteins Quantified in only one condition

Some proteins were quantified only in one condition. In such a case no p-values or fold change can be computed. Nevertheless, proteins with relatively high intensity in one condition but not present in the other condition can have biological relevance. Figure 13 shows how many protein were not quantified in a condition. The Figure 14 visualizes the most intensive proteins not quantified in the other condition.

Furthermore, in order to integrate those proteins with proteins which do have a fold change and a q.value, we also provide and a fold change estimate and q.value for those proteins. To emphasize that these values were not obtained by means of fitting a statistical model we call the columns in the output file which do contain them `pseudo.effectSize` and `pseudo.q.mod`. The reported pseudo fold change is computed by subtracting from the quantification value the average of the 10% smallest protein averages of the other condition while the q-value for those proteins was set to 0. The Volcano plot in Figure 15 envisages all the proteins quantified including those identified only in one of the samples (green).

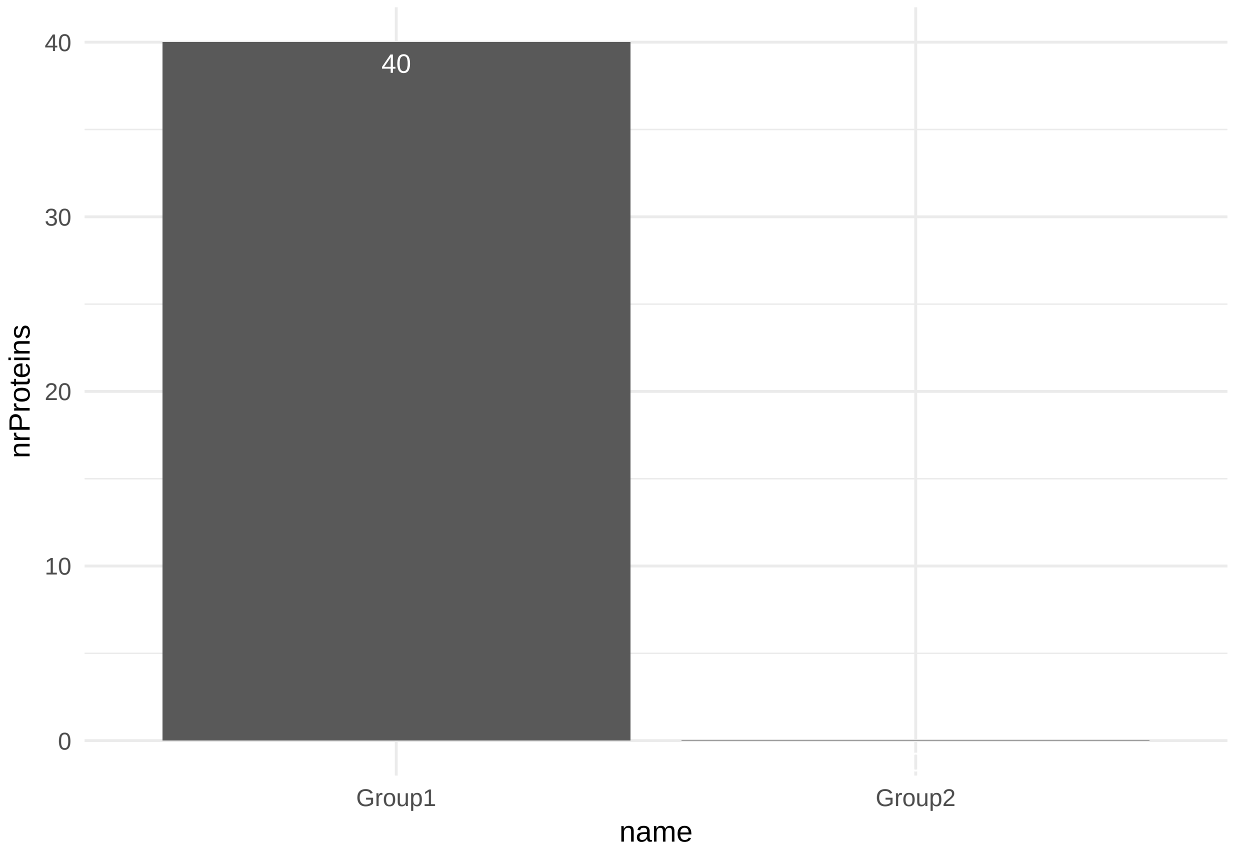

Figure 13: Nr of NAs in conditions.

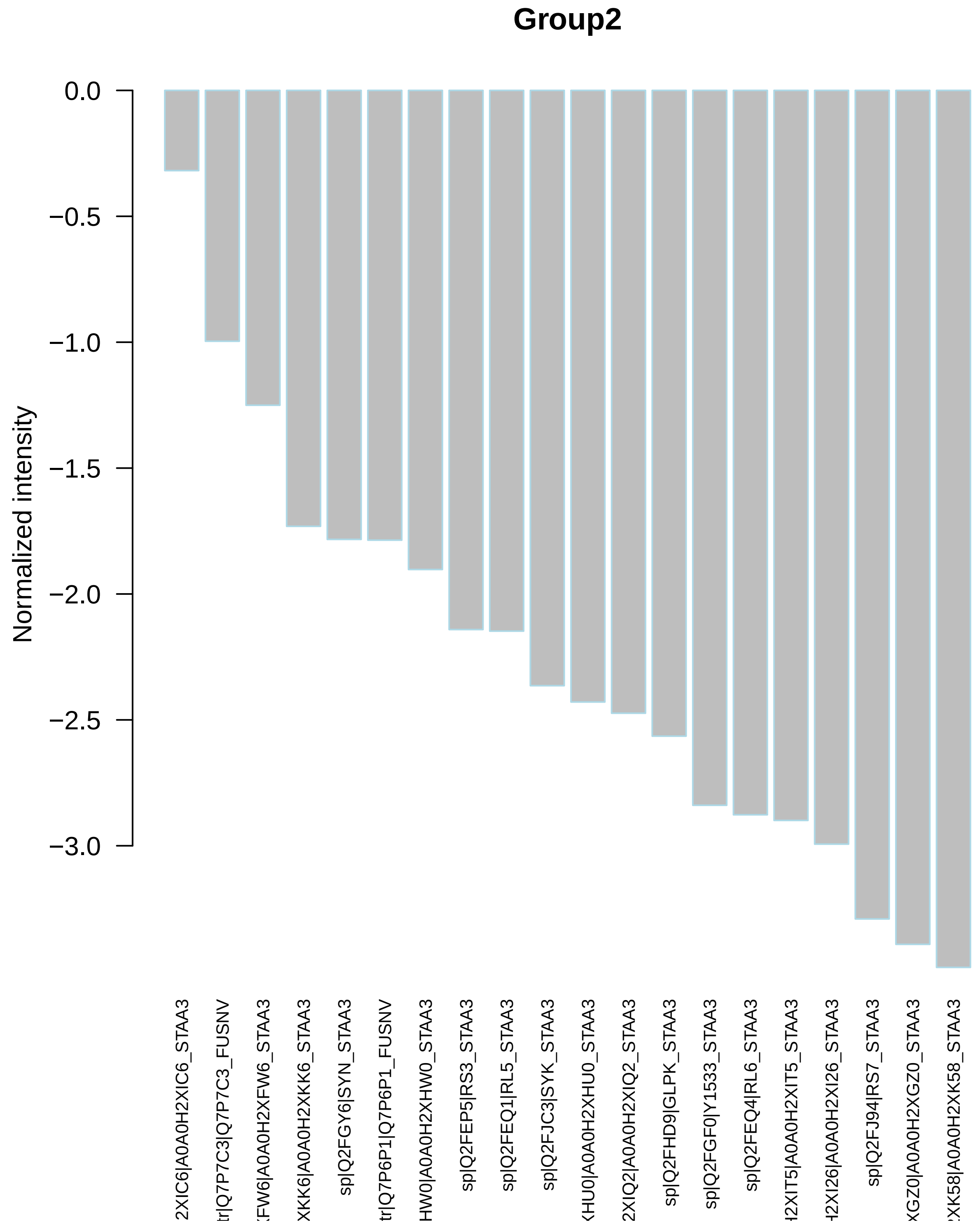

Figure 14: Proteins having a high normalized intensity in one condition not quantified in the second condition.

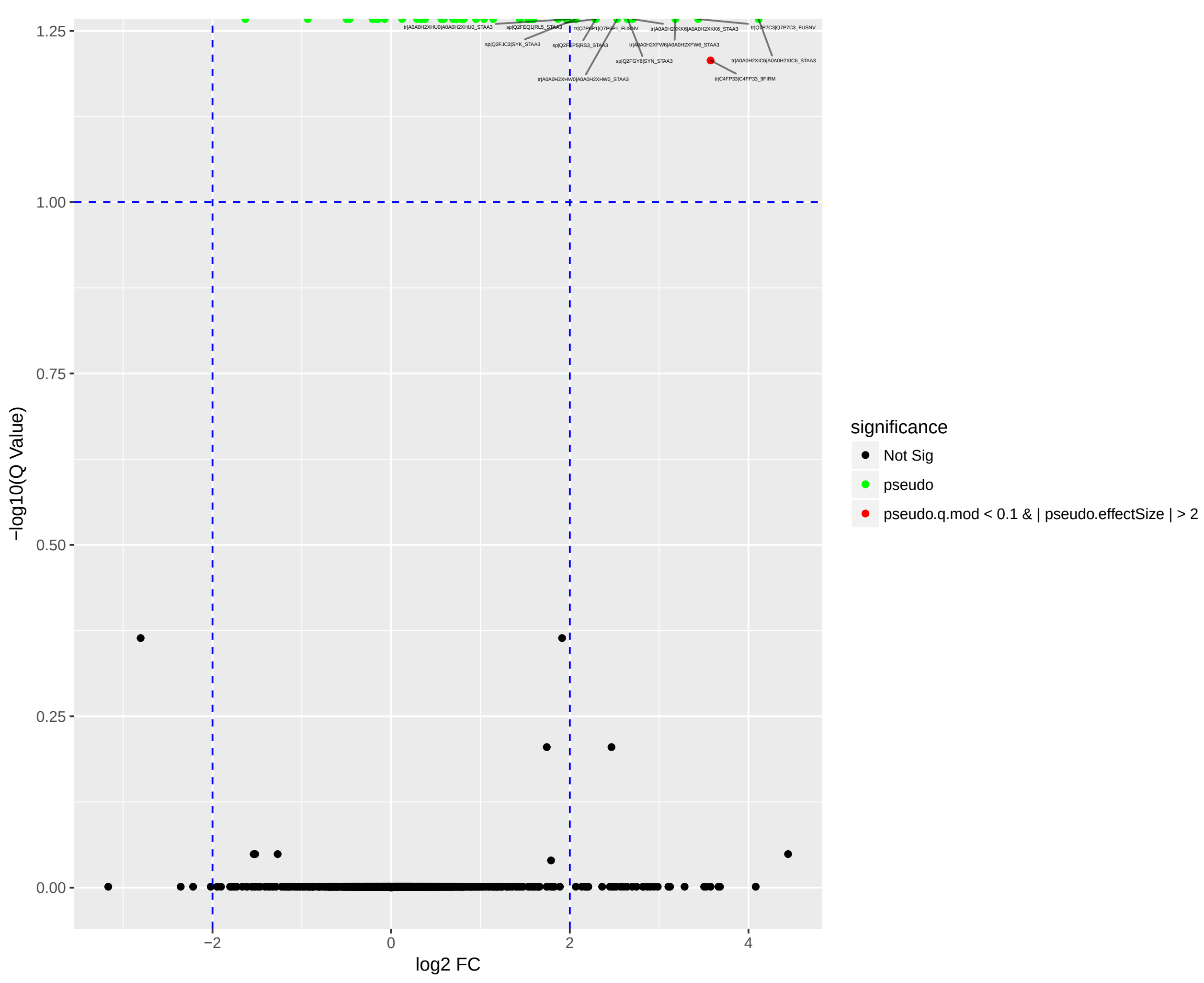

Figure 15: VolcanoPlot: x axis log2 fold change including pseudo log2 foldchanges of normalized data, y axis -log10(Q-value) inluding pseudo q-values.

3.3 List of Columns in the output table

columns
ProteinName
TopProteinName
nrPeptides
Fasta.headers
Group1
Group2
effectSize
p.ord
p.mod
q.ord
q.mod
log2FC
nrNAs
06_6_species_1.raw
10_6_species_2.raw
15_6_species_3.raw
08_6_sp_MSCRAMMs_3.raw
09_6_sp_MSCRAMMs_2.raw
14_6_sp_MSCRAMMs_1.raw
06_6_species_1.transformed
10_6_species_2.transformed
15_6_species_3.transformed
08_6_sp_MSCRAMMs_3.transformed
09_6_sp_MSCRAMMs_2.transformed
14_6_sp_MSCRAMMs_1.transformed
pseudo.Group1
pseudo.Group2
pseudo.effectSize
pseudo.q.mod

4 References

If you are going to use results produced by the scripts please do cite the SRMSerivce R package by providing the following URL www.github.com/protViz/SRMService by W.E. Wolski, J. Grossmann, C. Panse

For questions and improvements please do contact the authors of the package *SRMService*.

This report was generated using the package SRMService and quantable. The q-values and p-values were computed using the bioconductor package and limma.

4.1 Disclaimer

This document was generated using Rmarkdown and processes text files which are generated with a label-free quantitation software such as MaxQuant or Progenesis. The obtained results should be validated orthogonal as well (e.g. with Western blots). The Functional Genomics Center Zurich does not provide any kind of guarantee of the validity of these results.

5 Session Info

R version 3.4.3 (2017-11-30)

Platform: x86_64-pc-linux-gnu (64-bit)

locale: *LC*CTYPE=en_US.UTF-8_, *LC*NUMERIC=C_, *LC*TIME=en_US.UTF-8_, *LC*COLLATE=en_US.UTF-8_, *LC*MONETARY=en_US.UTF-8_, *LC*MESSAGES=en_US.UTF-8_, *LC*PAPER=en_US.UTF-8_, *LC*NAME=C_, *LC*ADDRESS=C_, *LC*TELEPHONE=C_, *LC*MEASUREMENT=en_US.UTF-8_ and *LC*IDENTIFICATION=C_

attached base packages: *stats*, *graphics*, *grDevices*, *utils*, *datasets*, *methods* and *base*

other attached packages: *rlang(v.0.2.0)*, *dplyr(v.0.7.5)*, *bindrcpp(v.0.2.2)*, *reshape2(v.1.4.2)*, *ggplot2(v.2.2.1)*, *quantable(v.0.3.4)*, *limma(v.3.30.13)*, *knitr(v.1.20)*, *bfabricShiny(v.0.9.15)*, *xml2(v.1.1.1)*, *shinyStore(v.0.1.0)*, *jsonlite(v.1.5)*, *httr(v.1.3.1)*, *PKI(v.0.1-3)*, *base64enc(v.0.1-3)*, *DT(v.0.2)* and *shiny(v.1.0.3)*

loaded via a namespace (and not attached): *tidyr(v.0.8.1)*, *splines(v.3.4.3)*, *foreach(v.1.4.4)*, *gtools(v.3.5.0)*, *assertthat(v.0.2.0)*, *highr(v.0.6)*, *stats4(v.3.4.3)*, *pander(v.0.6.1)*, *yaml(v.2.1.14)*, *ggrepel(v.0.6.5)*, *backports(v.1.0.5)*, *pillar(v.1.2.1)*, *lattice(v.0.20-35)*, *quantreg(v.5.33)*, *glue(v.1.2.0)*, *pROC(v.1.10.0)*, *digest(v.0.6.12)*, *RColorBrewer(v.1.1-2)*, *minqa(v.1.2.4)*, *colorspace(v.1.3-2)*, *htmltools(v.0.3.6)*, *httpuv(v.1.3.3)*, *Matrix(v.1.2-12)*, *plyr(v.1.8.4)*, *pkgconfig(v.2.0.1)*, *SparseM(v.1.77)*, *caret(v.6.0-76)*, *bookdown(v.0.4)*, *purrr(v.0.2.4)*, *xtable(v.1.8-2)*, *scales(v.0.5.0)*, *gdata(v.2.17.0)*, *lme4(v.1.1-13)*, *MatrixModels(v.0.4-1)*, *tibble(v.1.4.2)*, *mgcv(v.1.8-22)*, *car(v.2.1-4)*, *nnet(v.7.3-12)*, *lazyeval(v.0.2.0)*, *pbkrtest(v.0.4-7)*, *RJSONIO(v.1.3-0)*, *magrittr(v.1.5)*, *SRMService(v.0.1.8)*, *mime(v.0.5)*, *evaluate(v.0.10.1)*, *nlme(v.3.1-131)*, *MASS(v.7.3-47)*, *gplots(v.3.0.1)*, *class(v.7.3-14)*, *tools(v.3.4.3)*, *hms(v.0.3)*, *stringr(v.1.2.0)*, *munsell(v.0.4.3)*, *compiler(v.3.4.3)*, *e1071(v.1.6-8)*, *caTools(v.1.17.1)*, *grid(v.3.4.3)*, *nloptr(v.1.0.4)*, *iterators(v.1.0.9)*, *htmlwidgets(v.0.9)*, *labeling(v.0.3)*, *rmarkdown(v.1.9)*, *bitops(v.1.0-6)*, *gtable(v.0.2.0)*, *ModelMetrics(v.1.1.0)*, *codetools(v.0.2-15)*, *curl(v.2.6)*, *R6(v.2.2.2)*, *rprojroot(v.1.2)*, *bindr(v.0.1.1)*, *KernSmooth(v.2.23-15)*, *readr(v.1.1.1)*, *stringi(v.1.2.2)*, *parallel(v.3.4.3)*, *Rcpp(v.0.12.17)* and *tidyselect(v.0.2.4)*
